# CircLIFR synergizes with MSH2 to attenuate chemoresistance via MutSα/ATM-p73 axis in bladder cancer

**DOI:** 10.1186/s12943-021-01360-4

**Published:** 2021-04-19

**Authors:** Hui Zhang, Xingyuan Xiao, Wenjie Wei, Chao Huang, Miao Wang, Liang Wang, Yuanqiao He, Jiayin Sun, Yangkai Jiang, Guosong Jiang, Xiaoping Zhang

**Affiliations:** 1 Department of Urology, Union Hospital, Tongji Medical College, Huazhong University of Science and Technology, Wuhan, 430022 China; 2 Department of Laboratory Animal Science, Nanchang University, Nanchang, 330006 China

**Keywords:** Bladder cancer, CDDP, CircLIFR, MSH2

## Abstract

**Background:**

Cisplatin (CDDP) has become a standard-of-care treatment for muscle-invasive bladder cancer (MIBC), while chemoresistance remains a major challenge. Accumulating evidence indicates that circular RNAs (circRNAs) are discrete functional entities. However, the regulatory functions as well as complexities of circRNAs in modulating CDDP-based chemotherapy in bladder cancer are yet to be well revealed.

**Methods:**

Through analyzing the expression profile of circRNAs in bladder cancer tissues, RNA FISH, circRNA pull-down assay, mass spectrometry analysis and RIP, circLIFR was identified and its interaction with MSH2 was confirmed. The effects of circLIFR and MSH2 on CDDP-based chemotherapy were explored by flow cytometry and rescue experiments. Co-IP and Western blot were used to investigate the molecular mechanisms underlying the functions of circLIFR and MSH2. Biological implications of circLIFR and MSH2 in bladder cancer were implemented in tumor xenograft models and PDX models.

**Results:**

CircLIFR was downregulated in bladder cancer and expression was positively correlated with favorable prognosis. Moreover, circLIFR synergizing with MSH2, which was a mediator of CDDP sensitivity in bladder cancer cells, positively modulated sensitivity to CDDP in vitro and in vivo. Mechanistically, circLIFR augmented the interaction between MutSα and ATM, ultimately contributing to stabilize p73, which triggered to apoptosis. Importantly, MIBC with high expression of circLIFR and MSH2 was more sensitive to CDDP-based chemotherapy in tumor xenograft models and PDX models.

**Conclusions:**

CircLIFR could interact with MSH2 to positively modulate CDDP-sensitivity through MutSα/ATM-p73 axis in bladder cancer. CircLIFR and MSH2 might be act as promising therapeutic targets for CDDP-resistant bladder cancer.

**Supplementary Information:**

The online version contains supplementary material available at 10.1186/s12943-021-01360-4.

## Background

Bladder cancer is one of the most common cancer in the world and the most costly cancer to treat on a per patient basis due to required clinical surveillance and multiple therapeutic interventions [1]. Clinically, cisplatin (CDDP)-based gemcitabine and cisplatin (GC) regimen has become a standard-of-care treatment for muscle-invasive bladder cancer (MIBC) [2, 3]. Unfortunately, although 60% of patients with metastatic MIBC demonstrate an objective response to CDDP-based chemotherapy, this response is rarely durable, and chemoresistance remains a major challenge in this disease setting [2, 4]. More recently, immune checkpoint inhibitors (ICIs) have demonstrated robust evidence of therapeutic activity in metastatic MIBC [3, 5]. However, response rates from these uncontrolled immunotherapy trials are less than 30% [6]. Worse still, a retrospective cohort study shows decreased survival in patients treated with immunotherapy monotherapy relative to the chemotherapy arms [6]. In the management of MIBC, while combining ICIs with CDDP-based chemotherapy is an attractive approach, CDDP is still a first-line chemotherapeutic agent [3]. Thus, a better comprehension of the mechanisms underlying the development of CDDP resistance in patients with bladder cancer will represent a major step forward in optimizing patients’ outcomes.

The DNA mismatch repair (MMR) system guards against genomic instability, and mutations in the human MMR genes MutS homolog 2 (MSH2) and MutL homolog 1 (MLH1) are the cause of the majority of hereditary nonpolyposis colorectal cancer (HNPCC) [7]. In addition to the role in DNA repair, it is a somewhat unexpected finding that a major issue confronting the clinical management of tumors with MSH2 defects is that they are resistant to several of the common treatment regimes, such as CDDP [8–11]. In MSH2-deficient cells, DNA damage signaling involving p53 is suppressed during CDDP treatment in MEF cells [12]. Indeed, bladder tumors with low protein levels of MSH2 have poorer overall survival when treated with CDDP-base therapy, and the CDDP resistance screen suggests that MSH2 is the top one gene candidate based on statistical significance [13]. Due to the frequent mutation of TP53 in bladder cancer [14], the mechanism of MSH2 regulating chemotherapy resistance needs further study. On the other hand, it has been discovered that the interaction of MSH2 with other proteins is essential for triggering DNA damage signaling. Specifically, MSH2 interacts with MSH6 or MSH3 to form the MutSα or MutSβ complexes, respectively [15]. Nonetheless, intrinsic regulatory mechanisms of MSH2 affecting CDDP sensitivity remain largely unknown. Therefore, how to improve the chemosensitivity of bladder cancer with low expression of MSH2, as well as elucidating the underline mechanisms of MSH2-mediated CDDP sensitivity are of paramount importance.

Circular RNAs (circRNAs), which are a newly discovered class of non-coding RNAs (ncRNAs), are generated from back-splicing of pre-mRNAs to form covalently closed transcripts [16]. They were originally considered as erroneous products of splicing, but it has become clear that circRNAs are discrete functional entities [17, 18]. CircRNAs can serve as miRNA sponges to affect translational processing [19]. Additionally, circRNAs can interact with different proteins to form specific circRNA-protein complexes (circRNPs) that subsequently influence modes of action of associated proteins [20]. Notably, recent studies suggest an emerging picture of bladder cancer based on circRNAs, with unambiguous evidence of tumor promoting or inhibiting properties [21]. We recently found that circHIPK3, circNR3C1, BCRC-3 and has_circ_0001361 could affect the biological function by sponging miRNAs in bladder cancer [22–25]. Nevertheless, the regulatory functions as well as complexities of circRNAs in modulating CDDP resistance in bladder cancer are yet to be revealed.

In this study, we discovered that circLIFR, a circRNA generated from the circularization of LIFR gene, was significantly downregulated in bladder cancer. Subsequent studies showed that circLIFR could interact with MSH2 to positively modulate CDDP-sensitivity through MutSα/ATM-p73 axis in bladder cancer cell lines. Importantly, by using patient-derived xenograft (PDX) model, we further revealed that MIBC with high circLIFR and MSH2 levels were more sensitive to CDDP. Our findings provided a systematic elucidation into the regulation of circLIFR on the function of MSH2, and indicated that circLIFR and MSH2 might be act as promising therapeutic targets for CDDP-resistant bladder cancer.

## Methods

### Patients and tissue specimen collection

Seventy-nine pairs of bladder cancer tissues and paired adjacent normal bladder tissues were obtained from patients who underwent radical cystectomy at Department of Urology of the Union Hospital of Tong Medical College (Wuhan, China) between January 2015 and March 2019. With the instruction of a skillful pathologist, we collected the normal bladder urothelium samples (≥ 200 mg/sample) with a distance of ≥3 cm from the edge of cancer tissues in the resected bladder. All specimens were immediately snap-frozen in liquid nitrogen after surgical removal. Histological and pathological diagnoses were confirmed, and the specimens were classified by at least two experienced clinical pathologists according to the 2004 World Health Organization Consensus Classification and Staging System for bladder neoplasms. All specimens were obtained with appropriate informed consent from the patients and approved by the Institutional Review Board of Tongji Medical College of Huazhong University of Science and Technology. Detailed information is presented in Table 1. All of the patients were followed up on a regular basis, overall survival (OS) time was determined from the date of surgery to the date of death or the date of the last follow-up visit for survivors.

**Table 1:** Clinicopathological features of 79 bladder cancer patients and the expressions of circLIFR

Parameters	Group	Cases	circLIFR expression		*P* value
			High	Low	
Gender	Male	66	34	32	0.7695
	Female	13	6	7	
Age at surgery	< 55	11	5	6	0.7555
	≥55	68	35	33	
Pathological stage	pTa-T1	21	16	5	0.0100
	pT2-T4	58	24	34	
Lymph node metastasis	Absent	69	37	32	0.1927
	Present	10	3	7	
Vascular invasion	Absent	72	36	36	> 0.9999
	Present	7	4	3	
Muscle invasion	NMIBC	31	21	10	0.0210
	MIBC	48	19	29	
Total		79	40	39	

*p* < 0.05 represents statistical significance (Fisher’s exact test)

### Cell culture and treatment

Human invasive bladder cancer cell lines T24 (HTB-4) and UMUC3 (CRL-1749), human immortalized uroepithelium cell line SV-HUC-1 (CRL-9520), were purchased from American Type Culture Collection (ATCC, USA). UROtsa cells were generously provided by Drs. Donald and Maryann Sens (University of North Dakota, Grand Forks, ND). T24 and UMUC3 cells were maintained in Dulbecco’s modified Eagle’s medium (DMEM) supplemented with 10% FBS (Gibco, Australia origin), 1% penicillin/streptomycin, and 2 mM L-glutamine (Life Technologies, Carlsbad, CA, USA). SV-HUC-1 cells were cultured in the F-12 K medium supplemented with 10% FBS, 1% penicillin/streptomycin. Culture conditions of UROtsa cells were as previously described by Wnek [26]. All cells were grown in a humidified incubator at 37 °C with 5% CO2. Cisplatin (Sigma-Aldrich) was solubilized in DMSO or PBS.

### Induction of cisplatin-resistance in T24 cells

Cisplatin-resistant variants of T24 (T24-CDDP) were derived from original parental cell line by continuous exposure to cisplatin (Sigma-Aldrich, UK). Initially, T24 cells were treated with cisplatin (IC50) for 72 h. The media was removed and cells were allowed to recover for a further 72 h. This development period was carried out for approximately 4 months, after which time IC50 concentrations were re-assessed in resistant cell line. Cells were then maintained continuously in the presence of cisplatin at new IC50 concentration for a further 4 months.

### RNA preparation, RNase R, and qRT–PCR

Total RNA was isolated from cells or tissues using miRNeasy Mini Kit (Qiagen). Nuclear and cytoplasmic RNA was extracted using nuclear and cytoplasmic RNA purification kit (Fisher scientific, AM1921). For RNase R treatment, 1 μg of total RNA was incubated 15 min at 37 °C with or without 3 U of RNase R (Epicentre Technologies, Madison, WI). To validate backspliced junction point of circRNAs, the total RNA samples were treated with the RiboZero rRNA Removal Kit (Epicentre, WI, USA) for deleting rRNA, according to the manufacturer’s instructions; next, the rRNA depleted and RNase R digested RNA samples were synthesized cDNA with random primer (Takara, Dalian, China). To quantify the amount of mRNA and circRNA, cDNA was synthesized with the PrimeScript RT Master Mix (Takara, Dalian, China) from 500 ng of RNA. The real-time PCR analyses were performed using SYBR Premix Ex Taq II (Takara). In particular, the divergent primers annealing at the distal ends of circRNA were used to determine the abundance of circRNA. The primers are listed in Supplementary Table 1. Amplification was performed using the StepOnePlus Real-Time PCR System (Applied Biosystems, Foster City, CA) and Ct thresholds were determined by the software.

### Actinomycin D treatment and RNA stability assay for RNA lifetime

For actinomycin D treatment, cells were planted into six-well plates. Up to 60% confluency after 24 h, cells were treated with 5 μg/ml Actinomycin D or DMSO and collected at indicated time points.

The turnover rate and half-life of RNA was estimated according to a previously published paper [27]. As actinomycin D treatment results in transcription stalling, the change of RNA concentration at a given time (dC/dt) is proportional to the constant of RNA decay (*K*_decay_) and the RNA concentration (*C*), leading to the following equation:

$$ \mathrm{dC}/\mathrm{dt}=-{K}_{\mathrm{decay}}C $$

Thus, the RNA degradation rate *K*_decay_ was estimated by:

$$ \ln \left(C/{C}_0\right)=-{K}_{\mathrm{decay}}t $$

To calculate the RNA half-life (*t*_*1/2*_), when 50% of the RNA is decayed (that is, *C/C0* = 1/2), the equation was:

$$ \ln\ \left(1/2\right)=-{K}_{\mathrm{decay}}{t}_{1/ 2} $$

From where:

$$ {t}_{1/ 2}=\mathrm{ln}2/{K}_{\mathrm{decay}} $$

### RNA pull-down assays

Biotin-labelled circLIFR (sense) and control (antisense) probes (Supplementary Table 1) were synthesized by TSINGKE (Wuhan, China). RNA pull-down assays were performed as described [20]. In brief, 10^7^ cells were washed in ice-cold phosphate-buffered saline, lysed in 500 μl Co-IP buffer (20 mM Tris-HCL, pH 7.5, 150 mM NaCl, 1 mM EDTA, 0.5% NP-40, and complete protease inhibitors cocktail and RNase inhibitors), and incubated with 3 μg biotinylated DNA oligo probes, at room temperature for 2 h. A total of 50 μl washed Streptavidin C1 magnetic beads (Invitrogen) were added to each binding reaction and further incubated at room temperature for another hour. The beads were washed briefly with Co-IP buffer for five times. The bound proteins in the pull-down materials were analyzed by mass spectrometry or western blotting.

### Silver staining and mass spectrometry analysis

Silver staining was performed using the PAGE Gel Silver Staining Kit (Solarbio, Beijing, China) as the protocol described, while mass spectrometry analysis was done by Novogene (Tianjin, China). Afterwards, protein identification and quantification were accomplished by Proteome Discoverer software (version 1.4; Thermo Fisher Scientific, USA).

### Western blot and Immunoprecipitation

Whole cell lysates were prepared using RIPA buffer containing protease inhibitors (Beyotime, Shanghai, China). After boiling, the supernatants were subjected to SDS-PAGE and transferred to nitrocellulose membranes. After blocking with 5% non-fat milk, membranes were successively incubated with primary and HRP-conjugated secondary antibodies before visualizing bands using enhanced chemiluminescence (E412–01, Vazyme, Nanjing, China). For immunoprecipitation, cells were lysed in Co-IP buffer supplemented with protease inhibitor cocktail for 40 min on ice. Cell lysates were incubated with the indicated antibodies adsorbed to protein A/G Agarose (Thermo Fisher Scientific, #20421) for 4 h at 4 °C before washing three times in Co-IP buffer and elution at 95 °C for 10 min.

Antibodies used included primary antibodies against MSH2 (ab70270, Abcam, Cambridge, UK), AGO2 (Cell signaling technologies, #2897), MSH3 (22393–1-AP, Proteintech, USA), MSH6 (18120–1-AP, Proteintech, USA), ATM (ab78, Abcam, Cambridge, UK), pATM (ab81292, Abcam, Cambridge, UK), ATR (ab2905, Abcam, Cambridge, UK), p73 (ab137797, Abcam, Cambridge, UK), p63 (ab32353, Abcam, Cambridge, UK), α-tubulin (ab176560, Abcam, Cambridge, UK), histone H3 (ab52866, Abcam, Cambridge, UK), and GAPDH (60004–1-Ig, Proteintech, USA); HRP-conjugated secondary goat anti-mouse (SA00001–1), goat anti-rabbit (SA00001–2), HRP-mouse anti-rabbit IgG heavy chain specific (SA00001-7H) or HRP-mouse anti-rabbit IgG light chain specific (SA00001-7 L) antibodies (Proteintech, USA).

### RNA Immunoprecipitation (RIP)

RIP experiments were performed by using the Magna RIP RNA-Binding Protein Immunoprecipitation Kit (Millipore, Bedford, MA). Approximately 10^7^ cells were pelleted and re-suspended with an equal pellet volume of RIP Lysis Buffer (about 100 μl) plus protease and RNase inhibitors. The cell lysates (100 μl) were incubated with 5 μg of AGO2 (Cell signaling technologies, #2897), MSH2 (ab70270, Abcam, Cambridge, UK), or control Rabbit IgG (Thermo Fisher Scientific, #31235) coated beads with rotation at 4 °C overnight, respectively. After treating with proteinase K, the immunoprecipitated RNAs were extracted by RNeasy MinElute Cleanup Kit (Qiagen) and reversely transcripted using PrimeScript RT Master Mix (TaKaRa).

### Fluorescent in situ hybridization (FISH)

Cy3-labelled circLIFR probes (Supplementary Table 1) were synthesized by TSINGKE (Wuhan, China) and circLIFR FISH was performed as described with minor modifications [28]. Briefly, cells were fixed with the fixative solution, followed by permeabilization. Then hybridization was performed at 37 °C overnight in a dark moist chamber. After being washed three times in 2 × SSC (Solarbio, Beijing, China) for 10 min, the coverslips were sealed with parafilm containing DAPI. The images were acquired using a confocal laser scanning microscope (LSM 780, Carl Zeiss).

### Immunofluorescence

Bladder cancer cells grown on the coverslips were fixed with 4% paraformaldehyde in PBS for 20 min on ice and then permeabilized with 0.1% TritonX-100 in PBS for 10 min. After washing twice with PBS, cells were blocked with 5% BSA for 30 min at 37 °C and incubated with MSH2 antibody overnight at 4 °C. The next day, cells were washed with PBS and then incubated with corresponding secondary antibody for 30 min at 37 °C, followed by sealing with parafilm containing DAPI. Fluorescent images were acquired using a confocal laser scanning microscope (LSM 780, Carl Zeiss).

### Vector construction and cell transfection

To construct circLIFR and MSH2 over-expression plasmids, human circLIFR, MSH2 and p73 cDNAs were synthesized by TSINGKE (Wuhan, China) and cloned into pcDNA3.1(+) CircRNA Mini Vector (addgene #60648) and p3XFLAG-CMV-10 vector (Sigma-Aldrich), respectively. Truncations of MSH2 were amplified with primers (Supplementary Table 1), and subcloned into p3XFLAG-CMV-10 vector. Oligonucleotides encoding short hairpin RNAs (shRNAs) specific for circLIFR, MSH2, and p73 (Supplementary Table 1) were cloned into pLKO.1-puro (Sigma-Aldrich). Transfection was carried out using Lipofectamine 2000 (Life Technologies) according to the manufacturer’s instructions. Stable cell lines were screened by administration of neomycin or puromycin (Invitrogen). Empty vector and scramble shRNA (sh-Scb) were applied as controls (Supplementary Table 1).

### Nuclear and cytoplasmic extraction

Cytoplasmic and nuclear fractions were isolated as described by the manufacturer, using the reagents supplied in PARIS™ Kit (AM1556, Thermo Fisher Scientific, Waltham, USA). Briefly, cells were lysed in Cell Fraction Buffer on ice for 10 min. After centrifugation at 500×g for 3 min at 4 °C, the supernatant was collected as cytoplasmic fraction. Followed by washing the pellet with Cell Fraction Buffer, the nuclei were collected.

### Gene set enrichment analysis (GSEA)

Gene set enrichment analysis was performed as previously described [29]. The published gene sets were used as indicated. Datasets were generated from TCGA database [30].

### Cell counting Kit-8 (CCK-8) assay

The proliferation of cells was tested by CCK-8 kit (Dojindo, Japan) following the manufacturer’s instructions. The optical density at 450 nm was measured using an automatic microplate reader (Synergy4; BioTek, Winooski, VT, USA).

### Apoptosis assay

For the apoptosis assay, cells were seeded into a six-well plate with or without CDDP treatment. The cell apoptosis assay was determined according to the manual of FITC Annexin V Apoptosis Detection Kit I (BD Biosciences). Data were analyzed by FlowJo software (FlowJo).

### Tumor xenograft model

All animal experiments were approved by the Animal Care Committee of Tongji Medical College. The BALB/c nude mice (4 weeks old, ♀) were obtained from Beijing Vital River Laboratory Animal Technology Co., Ltd. and housed in a specific pathogen free facility. Cells were injected subcutaneously into the dorsal flanks of nu/nu mice (3 × 10^6^ cells per mouse). Tumors were measured with calipers and calculated using the following formula: a^2^ × b × 0.5, where a is the smallest diameter and b is the diameter perpendicular to a. At the end of the experiment, mice were sacrificed and tumors were excised and weighed.

For orthotopic bladder tumor model, the experiments were performed as described previously with minor modifications [31]. In brief, under anesthesia, the nu/nu mice were placed in a supine position on a thermostatic blanket and urethras were catheterized with 18G intravenous. Silver nitrate was injected and allowed to dwell for 10 s, following bladder irrigation by injecting sterile water. Then, prepared 2 × 10^6^ cells were injected using the stylet needle. Tumors were monitored by ultrasound imaging twice a week.

For in vivo drug studies, CDDP or PBS was administered by intraperitoneal injection three-times weekly at the dose of 1 mg/kg.

### Patient-derived xenograft model

The effects of circLIFR and MSH2 were evaluated by using the widely accepted patient-derived xenograft (PDX) model. The tumors were removed and cut into small pieces with a volume of 30–60 mm^3^ when grown to ~ 800 mm^3^, and subcutaneously inoculated into the flanks of the NOD-SCID mice. The tumor xenografts were used for experiments after three serial passages. Tumor pieces of ~ 60 mm^3^ were subcutaneously grafted into the flanks of the NOD-SCID mice. When tumors grown to ~ 200 mm^3^, the mice were randomly divided into PBS or CDDP subgroups. After that, mice with tumors were injected intraperitoneally with either PBS, or CDDP (2 mg/kg) at day 1, 2, 3, 15, 16, and 17. Tumor growth was assessed with caliper every 3 to 4 days. Tumor volumes were measured using the following formula: 4π / 3 × (width / 2)^2^ × (length / 2). At day 28, animals were sacrificed under anesthesia, after which tumors were harvested and immediately snap-frozen in cold 2-methylbutane.

### Histology, immunohistochemistry (IHC), and terminal deoxyribonucleotide transferase-mediated nick-end labeling (TUNEL) analyses

Tumor tissues were fixed in 4% paraformaldehyde (PFA) and embedded in paraffin. 4-μm sections were processed for IHC and TUNEL analyses. IHC analyses were performed with primary antibodies specific for MSH2 (ab70270, Abcam, Cambridge, UK), pATM (ab81292, Abcam, Cambridge, UK), p73 (ab137797, Abcam, Cambridge, UK), using procedures previously described [22]. For TUNEL, tissue slides were incubated with the TUNEL BrightGreen Apoptosis Detection Kit (Vazyme, Nanjing, China).

### Statistics

Data were expressed as Mean ± SD. Analyses were performed using Prism 8.1.2 (GraphPad Software Inc.). Mean of the groups were compared using a student t-test and ANOVA. Kaplan-Meier survival curves for mice and *P*-values were calculated using a log-rank test. *P* values of < 0.05 indicate statistical significance.

## Results

### Identification and characterization of circLIFR in bladder cancer

**Fig. 1:**
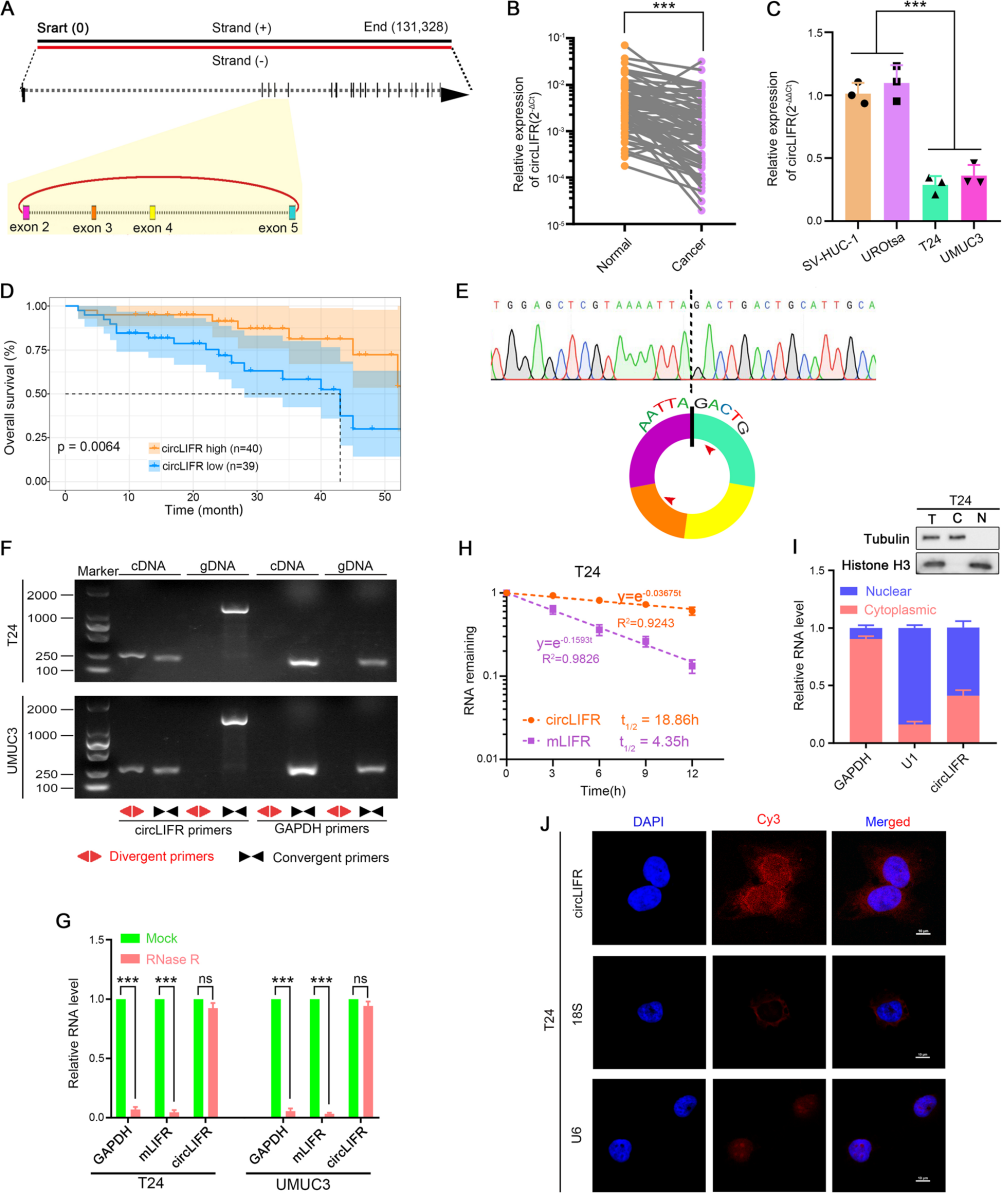
Identification and distribution of circLIFR. **a** Scheme illustrating the production of circLIFR. **b**, **c** The expression of circLIFR was detected by qRT-PCR in 79 pairs of bladder cancer and paired adjacent normal bladder tissues, SV-HUC-1, UROtsa, T24, and UMUC3 cells. GAPDH was used as internal control. Data were mean ± SD. ***P < 0.001 (Student’s t-test). **d** Kaplan-Meier curves of OS in bladder cancer patients. Patients were grouped by the median circLIFR expression. *P*-value was calculated using a log-rank test. **e** Sequencing analysis of head-to-tail splicing junction in circLIFR. **f** The existence of circLIFR was validated in T24 and UMUC3 bladder cancer cell lines by qRT-PCR. Divergent primers amplified circLIFR in cDNA but not genomic DNA (gDNA). GAPDH was used as negative control. Red arrows indicated divergent primers, and black arrows indicated convergent primers. **g** The relative RNA levels were analyzed by qRT-PCR in T24 and UMUC3 cells treated with or without RNase R. Data were mean ± SD, *n* = 3. ns, not significant, ***P < 0.001 (Student’s t-test). **h** The relative RNA levels of circLIFR and mLIFR were analyzed by qRT-PCR after treatment with actinomycin D at the indicated time points in T24 cells (*n* = 3). **i** Identification of circLIFR cytoplasmic and nuclear distribution by qRT-PCR analysis in T24 cells. GAPDH and U1 were applied as positive controls in the cytoplasm and nucleus, respectively (*n* = 3). Western blots of total cell lysates (T), cytosolic extracts (C) and nuclear extracts (N) with α-tubulin as a cytosolic marker, histone H3 as a nuclear marker. **j** Identification of circLIFR cytoplasmic and nuclear distribution by FISH in T24 cells. 18S and U6 were applied as positive controls in the cytoplasm and nucleus, respectively; circLIFR, 18S, and U6 probes were labeled with Cy3; nuclei were stained with DAPI

Previously, we have analyzed the expression profile of circRNAs in human bladder cancer tissues and paired normal tissues through high-throughput sequencing [22]. Among the differentially expressed circRNAs, we noted that hsa_circ_0072309 (termed as circLIFR) was derived from the exons 2, 3, 4 and 5 regions within the LIFR locus (Fig. 1a), utilizing the human reference genome (GRCh37/hg19). LIFR is a key gene in the pathogenesis of tumors of different histology [32–34]. Consistent with the RNA-seq results [22], circLIFR was significantly downregulated in bladder cancer tissues (Fig. 1b), while the expression of LIFR pre-mRNA (pLIFR) and mRNA (mLIFR) showed no significant difference between bladder cancer and paired normal tissues (Fig. S1, A and B). Down-regulation of circLIFR was also found in human muscle-invasive bladder cancer cells T24 and UMUC3, compared with human immortalized uroepithelium cells SV-HUC-1 and UROtsa (Fig. 1c). Moreover, Kaplan-Meier curves showed that low levels of circLIFR predicted a shorter survival times for overall survival (OS) (Fig. 1d), while similar survival times for OS were found between different expression levels of mLIFR (Fig. S1C). Therefore, these findings indicated that the lower expression of circLIFR in bladder cancer was not simply a by-product of splicing and was suggestive of functionality.

CircLIFR was a 580-nt circRNA, the backspliced junction point of which was amplified with divergent primers and validated by Sanger sequencing (Fig. 1e). To further confirm the circular characteristics of circLIFR, comparison of random 6 mers- versus oligo dT-primed cDNA synthesis was performed. It showed that circLIFR was retro-transcribed more efficiently with random 6 mers than with oligo dT primer, which indicated that circLIFR had no poly-A tail (Fig. S1D). Next, the head-to-tail splicing of endogenous circLIFR was assayed by RT-PCR with convergent and divergent primers. As expected, circLIFR could be amplified by the divergent primers in cDNA but not genomic DNA (gDNA) (Fig. 1f). Resistance to digestion with RNase R exonuclease also confirmed that circLIFR harbored a circular RNA structure (Fig. 1g). Moreover, circLIFR transcripts were more stable in comparison to LIFR mRNA upon treatment with actinomycin D (Fig. 1h and Fig. S1E). In addition, qRT-PCR analysis of the nuclear/cytoplasmic fractionation and fluorescence in situ hybridization (FISH) detection showed that circLIFR was mainly localized in the nucleus (Fig. 1i and j, and Fig. S1, F and G).

Collectively, these findings established that circLIFR was a bona fide circRNA, which was predominantly distributed in nucleus and was significantly downregulated in bladder cancer.

### CircLIFR interacts with MSH2 protein in bladder cancer cells

Similar to other ncRNAs, defining the subcellular localization of circRNAs could provide valuable insights into their functions. To determine whether cytoplasm-localized circLIFR functions as a miRNA sponge, we analyzed argonaut 2 (AGO2) CLIP and found that circLIFR did not bind to AGO2 [35], which was supported by an AGO2 reciprocal immunoprecipitation (RIP) assay (Fig. S2A). Thus, we ruled out the function of circLIFR that acted as miRNA sponge.

**Fig. 2:**
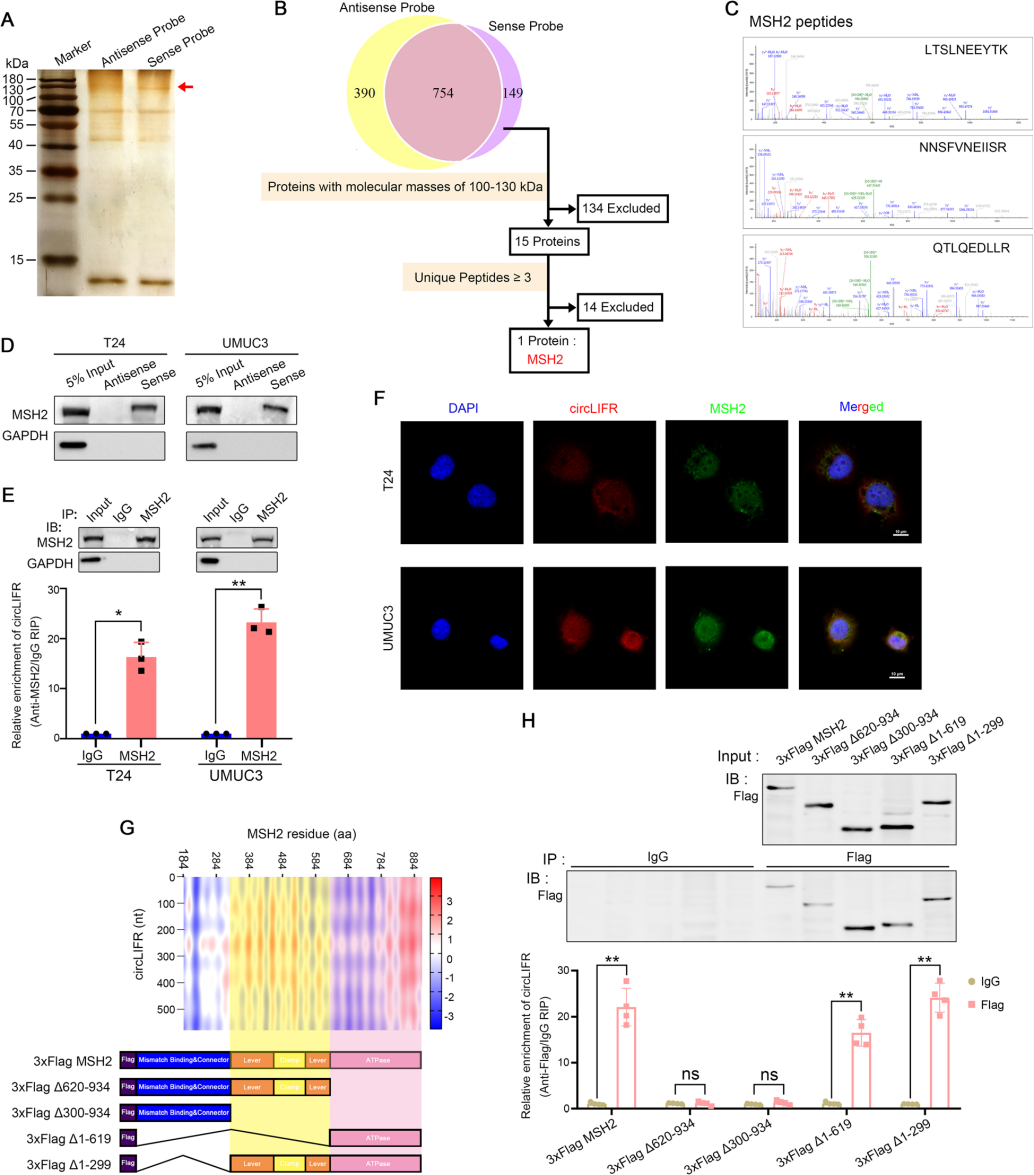
CircLIFR binds to MSH2 protein. **a** Biotin-labeled sense or antisense circLIFR probes were used for RNA-protein pull-down against T24 cell lysates. Identification of proteins that interact with circLIFR by silver staining. Red arrow indicates the major differential band precipitated in T24 lysates. **b** Analysis pipeline was performed to identify proteins that interact with circLIFR: (1) The 149 proteins that were only pulled down by sense probe were screened; (2) The 15 proteins with molecular masses of 100–130 kDa were then selected as the candidates according to the positive band found in silver staining; (3) MSH2 was selected as it was the only protein with high abundance (no less than 3 peptides). **c** Mass spectrometry assay depicted the MSH2 peptides pulled down by sense circLIFR probes. **d** MSH2 immunoblot analysis of the biotin-labeled sense and antisense circLIFR probes pull-down eluate from lysates of T24 and UMUC3 cells. GAPDH was used as loading control. **e** RNA immunoprecipitation (RIP) assays in T24 and UMUC3 cells using MSH2 and IgG antibody. The precipitate was subjected to western blotting with the antibodies against MSH2 and GAPDH. The MSH2-enriched circLIFR relative to the IgG-enriched value was calculated by qRT-PCR. Data were mean ± SD. *P < 0.05, **P < 0.01 (Student’s t-test). **f** Dual RNA-FISH and immunofluorescence staining assay indicating the co-localization of circLIFR (red) and MSH2 (green), with nuclei staining with DAPI (blue). **g** Prediction of circLIFR-MSH2 interaction by using the catPAPID algorithm and schematic of MSH2 with functional protein domains. MSH2 truncations lacking the region 620–934 aa (3xFlag Δ620–934), 300–934 aa (3xFlag Δ300–934), 1–619 aa (3xFlag Δ1–619), or 1–299 aa (3xFlag Δ1–299). **h** Relative enrichment of endogenous circLIFR in truncated MSH2 RIP was measured by qRT-PCR, following T24 cells transfected with 3xFlag-MSH2 truncations. Data were mean ± SD. ns, not significant, **P < 0.01 (Student’s t-test)

Given that circLIFR mainly located in the nucleus, we next performed RNA pulldown assays to explore its protein binding role, using biotinylated probes targeting the circLIFR back-spliced sequence (Fig. 2a and Fig. S2B). Following the analysis pipeline (Fig. 2b and Supplementary Table 2 and 3) to identify RBPs, a major differential band precipitated in T24 lysates was identified to be MSH2 through mass spectrometry (Fig. 2c). The interaction between circLIFR and MSH2 was further validated through probing the precipitates immunoprecipitated by anti-MSH2 antibody (Fig. 2d) and RIP analysis (Fig. 2e). Furthermore, we confirmed the colocalization of endogenously expressed circLIFR and MSH2 in the nucleus by performing immunofluorescence and fluorescence in situ hybridization assays (Fig. 2f).

To delineate the structural determinants of the interactions between circLIFR and MSH2, we carried out deletion mapping by subdividing the MSH2 functional domains. Using catRAPID algorithm for RNA-protein interaction [36], circLIFR was predicted to bind with the lever, clamp and ATPase domains of MSH2 protein (Fig. 2g). An anti-Flag RIP assay showed that removal of the ATPase domain (aa620–934) of MSH2, which domain is intrinsically linked to conformational changes of MMR proteins [10, 37–39], abolished its association with circLIFR, while deletion of the lever and clamp domains (aa300–620) had no effect on its interaction with circLIFR (Fig. 2h). In summary, these results proposed that circLIFR/MSH2 formed an RNA-protein complex through the ATPase domain of MSH2 in bladder cancer cells.

### MSH2 is a mediator of up-regulation of CDDP sensitivity in bladder cancer cells

**Fig. 3:**
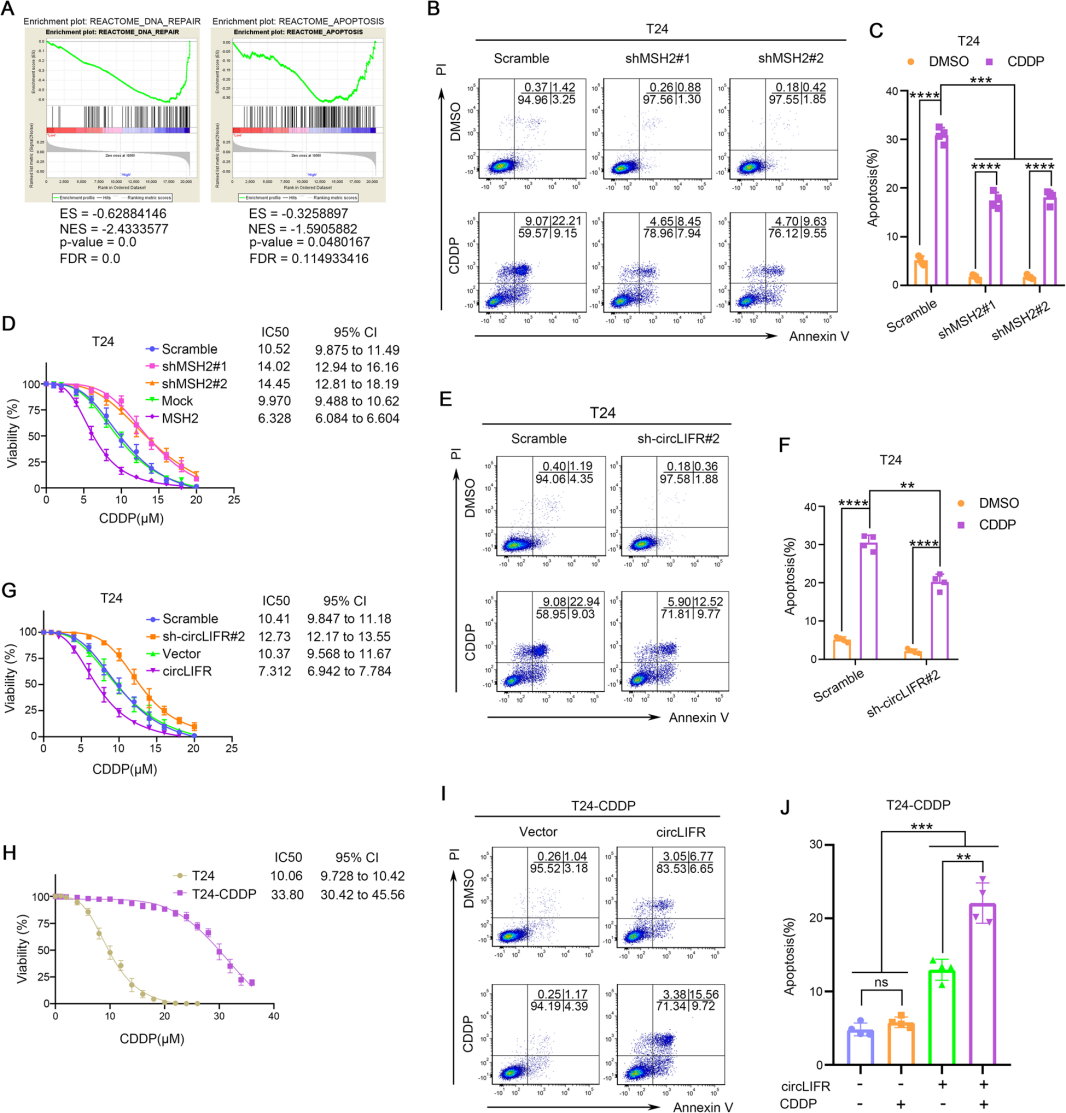
MSH2 and circLIFR can improve CDDP chemosensitivity. **a** Gene set enrichment analysis (GSEA) of TCGA datasets showed that higher MSH2 expression was significantly associated with DNA repair and apoptosis in bladder cancer. **b**, **c** T24 cells were stably transfected with scramble, shMSH2#1, or shMSH2#2 vector. After T24 cells were treated for 36 h in the absence or presence of 5 μM CDDP, apoptosis was measured by Annexin-V plus PI staining and fluorescence-activated cell sorter (FACS) analysis. Bars show the percentages of cells that were early apoptotic (Annexin-V^+^/PI^−^) and late apoptotic/dead (Annexin-V^+^/PI^+^). Data were mean ± SD. ***P < 0.001, ****P < 0.0001 (Student’s t-test). **d** Determination of IC50 values for CDDP treatment 24 h in T24 cells which were stably transfected with scramble, shMSH2#1, shMSH2#2, mock, or MSH2 vector. **e**, **f** T24 cells were stably transfected with scramble, sh-circLIFR#2 vector. After T24 cells were treated for 36 h with or without 5 μM CDDP, apoptosis was measured by Annexin-V plus PI staining and FACS analysis. Data were mean ± SD. **P < 0.01, ****P < 0.0001 (Student’s t-test). **g** Determination of IC50 values for CDDP treatment 24 h in T24 cells which were stably transfected with scramble, sh-circLIFR#2, vector, or circLIFR. **h** Determination of IC50 values for CDDP treatment 24 h in T24 and T24-CDDP cells. **i**, **j** T24-CDDP cells were stably transfected with vector or circLIFR. After T24-CDDP cells were treated for 36 h in the absence or presence of 5 μM CDDP, apoptosis was measured by Annexin-V plus PI staining and FACS analysis. Data were mean ± SD. ns, not significant, **P < 0.01, ***P < 0.001 (Student’s t-test)

It was previously reported that MSH2 could not only protect mammalian genomes by repairing mismatched bases resulted from erroneous DNA replication, but also promote apoptosis as part of the cellular response to CDDP [10, 11, 40]. Recent study indicated that bladder cancer cells depleted of MSH2 were resistant to CDDP in vitro, in part due to a reduction in p53-dependent apoptosis [13]. However, the role of MSH2 in CDDP-based chemotherapy, especially in p53-deficient bladder cancer, remains to be further investigated. In this regard, we explored whether MSH2 played a vital role in CDDP resistance in T24 and UMUC3 bladder cancer cell lines, which are p53-deficient cells (Supplementary Table 4). Gene set enrichment analysis (GSEA) indicated that MSH2 was highly associated with DNA repair and apoptosis based on the data from TCGA database (Fig. 3a). Knockdown of MSH2 markedly decreased the apoptosis rate in T24 and UMUC3 cells treated with CDDP (Fig. 3b and c, and Fig. S3, A to C). Moreover, our results showed that IC50 value of CDDP was increased when MSH2 was knocked down, and decreased when MSH2 was overexpressed (Fig. 3d and Fig. S3D). Collectively, our findings demonstrated that MSH2 was a mediator of up-regulation of CDDP sensitivity through inducing apoptosis in p53-deficient bladder cancer cells.

### CircLIFR positively modulates sensitivity of bladder cancer cells to CDDP

Given that circLIFR could interact with MSH2 to form RNA-protein complex, we subsequently evaluated the potential effect of circLIFR on CDDP sensitivity in bladder cancer cells. First, the fidelity of the knockdown and overexpression systems used to manipulate circLIFR expression was evaluated. CircLIFR knockdown experiments with independent small hairpin RNAs (shRNAs) designed against back-splicing between exons 2 and 5 of circLIFR revealed that sh-circLIFR#2 could specifically target circLIFR, but not mLIFR (Fig. S3E). Meanwhile, overexpression of circLIFR was confirmed to have no effect on expression of mLIFR (Fig. S3F). Next, we observed that silencing of circLIFR decreased CDDP-induced apoptosis in T24 and UMUC3 cells (Fig. 3e and f and Fig. S3, G and H). Moreover, as determined by CCK8 assay, CDDP sensitivity was enhanced upon overexpression of circLIFR, and was decreased after knockdown of circLIFR in bladder cancer cells (Fig. 3g and Fig. S3I). We then sought to define whether circLIFR was effective against acquired CDDP resistance in bladder cancer. To this end, we continuously exposed T24 cells to stepwise escalating concentrations of CDDP and established a CDDP resistant T24 cell line (named T24-CDDP). We confirmed that T24-CDDP resistant cells exhibited a high level of resistance to CDDP (Fig. 3h), while there was no significant difference in circLIFR levels and MSH2 mRNA/protein levels between T24-CDDP resistant and parental T24 cells (Fig. S3J). Importantly, overexpression of circLIFR could sensitize T24-CDDP resistant cells to CDDP-induced apoptosis (Fig. 3i and j). Altogether, we concluded that circLIFR promoted apoptosis and overcame acquired resistance of bladder cancer cells to CDDP in vitro, and might be a potential therapeutic target for CDDP resistance.

### CircLIFR/MSH2 complex contributes to the CDDP sensitivity via MutSα/ATM-p73 axis in bladder cancer cells

**Fig. 4:**
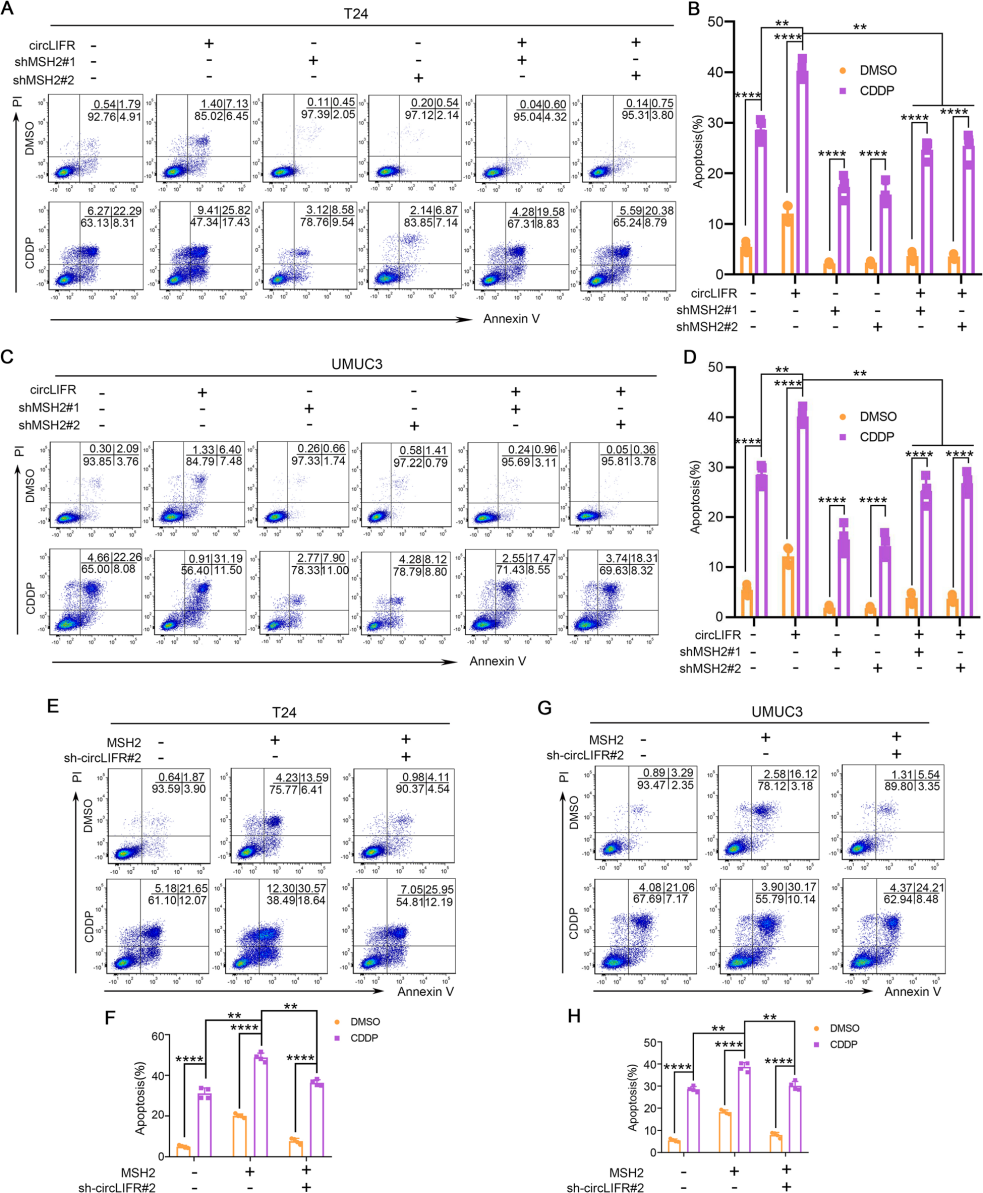
CircLIFR synergizes with MSH2 to enhance CDDP chemosensitivity of bladder cancer cells. **a**-**d** FACS assay showing the apoptosis of T24 and UMUC3 cells stably transfected with vector or circLIFR, and those cotransfected with scramble, shMSH2#1, or shMSH2#2. Data were mean ± SD. **P < 0.01, ****P < 0.0001 (Student’s t-test). **e**-**h** FACS assay showing the apoptosis of T24 and UMUC3 cells stably transfected with vector or MSH2, and those cotransfected with scramble, sh-circLIFR#2. Data were mean ± SD. **P < 0.01, ****P < 0.0001 (Student’s t-test)

To further determine the role of circLIFR and MSH2 complex in bladder cancer CDDP chemosensitivity, we performed MSH2 knockdown in circLIFR-overexpressed bladder cancer cells, and observed that circLIFR induction of cell apoptosis upon CDDP treatment was reversed by knockdown of MSH2 (Fig. 4a to d), suggesting that the up-regulation of cell apoptosis and CDDP sensitivity by circLIFR was dependent on its interaction with MSH2. On the other hand, we knocked down circLIFR in MSH2-overexpressed bladder cancer cells, and found that MSH2 promotion of cell apoptosis upon CDDP treatment was also down-regulated by knockdown of circLIFR (Fig. 4e to h). These results provided the evidences that circLIFR could synergize with MSH2 to enhance CDDP chemotherapeutic efficacy of bladder cancer cells.

**Fig. 5:**
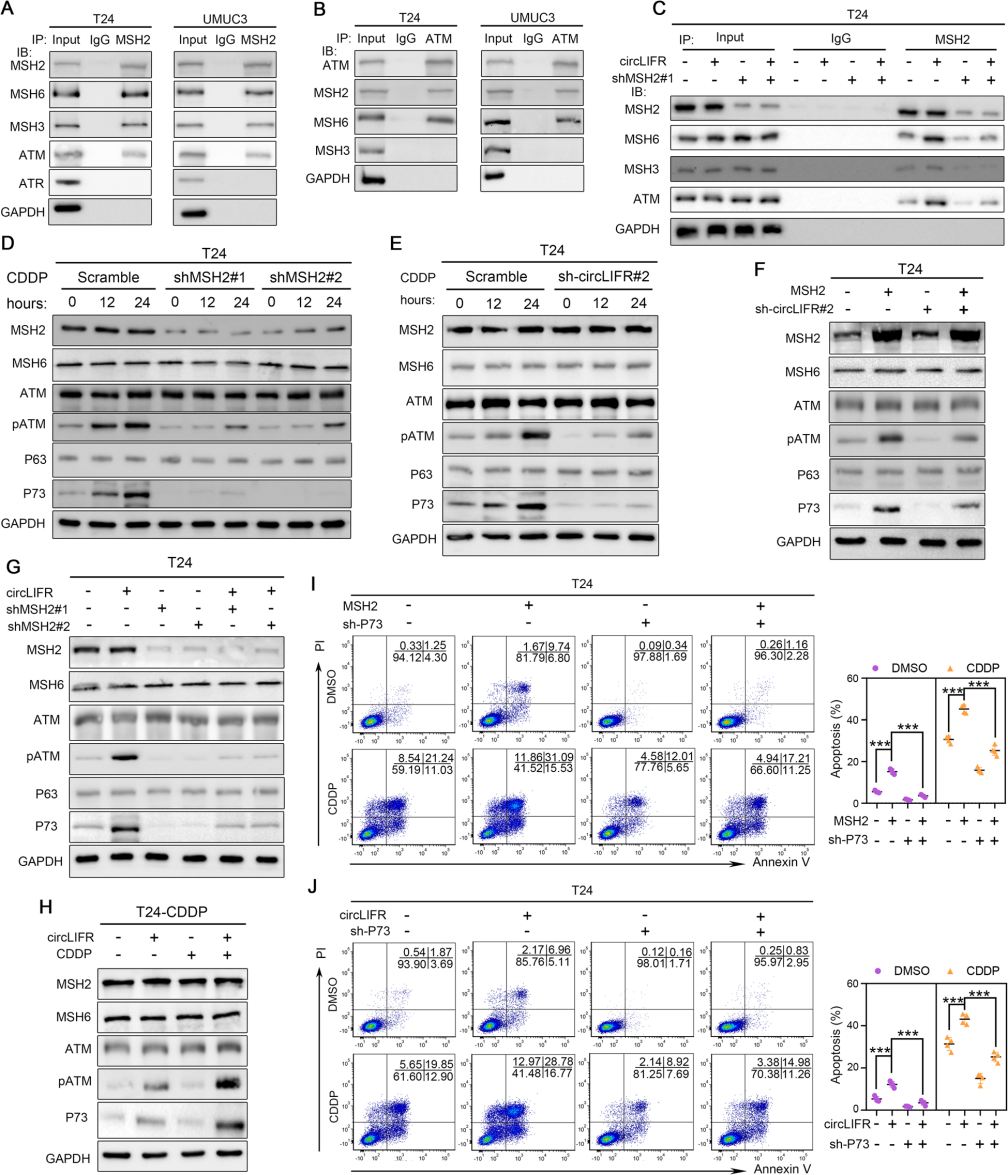
CircLIFR/MSH2 complex contributes to the CDDP sensitivity via MutSα/ATM-p73 axis in bladder cancer cells. **a** Co-IP assay was analyzed using T24 and UMUC3 cells lysate which was immunoprecipitated by anti-MSH2 antibody. The precipitate was subjected to western blotting with the antibodies against MSH2, MSH6, MSH3, ATM, ATR, and GAPDH. **b** Co-IP assay was analyzed using T24 and UMUC3 cells lysate which was immunoprecipitated by anti-ATM antibody. The precipitate was subjected to western blotting with the antibodies against ATM, MSH2, MSH6, MSH3, and GAPDH. **c** Interaction between MSH2, MSH6, MSH3, and ATM in T24 cells stably transfected with vector or circLIFR, and those cotransfected with scramble, or shMSH2#1. Co-IP experiments with anti-MSH2 antibody were performed, and the precipitate was detected by western blot with the antibodies against MSH2, MSH6, MSH3, ATM, and GAPDH. **d** T24 cells, which were stably transfected with scramble, shMSH2#1, or shMSH2#2, were treated with 5 μM CDDP for the indicated time. Whole cell lysates were collected for western blot analysis of MSH2, MSH6, ATM, pATM, p73, p63, and GAPDH. **e** T24 cells, which were stably transfected with scramble, or sh-circLIFR#2, were treated with 5 μM CDDP for the indicated time. Whole cell lysates were collected for western blot analysis of MSH2, MSH6, ATM, pATM, p73, p63, and GAPDH. **f** Western blot analysis with the indicated antibodies in T24 cells stably transfected with vector or MSH2, and those cotransfected with scramble, or sh-circLIFR#2. **g** Western blot analysis with the indicated antibodies in T24 cells stably transfected with vector or circLIFR, and those cotransfected with scramble, shMSH1#1, or shMSH1#2. **h** T24-CDDP cells stably transfected with vector or circLIFR were treated with or without 5 μM CDDP for 24 h, and cell lysates were subjected to western blot analysis with the indicated antibodies. **i** T24 cells were stably transfected with vector or MSH2 and cotransfected with scramble, or sh-p73. Apoptosis was measured by Annexin-V plus PI staining and FACS analysis. Data were mean ± SD. ***P < 0.01 (Student’s t-test). **j** T24 cells were stably transfected with vector or circLIFR and cotransfected with scramble, or sh-p73. Apoptosis was measured by Annexin-V plus PI staining and FACS analysis. Data were mean ± SD. ***P < 0.01 (Student’s t-test)

We then elucidated the molecular mechanism by which circLIFR/MSH2 complex contributed to CDDP sensitivity in bladder cancer cells. It showed that overexpression of circLIFR had no effect on both mRNA and protein levels of MSH2 (Fig. S4A). Meanwhile, we found no significant difference of circLIFR levels between MSH2-overexpressed cells and control cells (Fig. S4B). These results led us to speculate that circLIFR might regulate apoptosis and CDDP sensitivity by affecting the activity, rather than the abundance of MSH2 protein. As an obligate subunit for MMR proteins in eukaryotic cells, MSH2 interacts with MSH6 or MSH3 to form the MutSα or MutSβ complexes, respectively [15]. Furthermore, it has been demonstrated that MutSα forms a complex with ATM, which is well known for its role as an apical activator of the DNA damage response [41]. Therefore, we carried out co-immunoprecipitation with anti-MSH2 antibody, and it indicated that MSH2 existed as a stable complex with MSH6, MSH3 and ATM, but not ATR, in T24 and UMUC3 cells (Fig. 5a). Meanwhile, immunoprecipitation of ATM co-precipitated MutSα, but not MutSβ (Fig. 5b). We further observed that circLIFR overexpression enhanced the interaction of endogenous MSH2 with MSH6, which was attenuated upon MSH2 knockdown, while circLIFR had slight effect on the binding of MSH2 with MSH3 (Fig. 5c and Fig. S4C). Importantly, we also found that the association of MSH2 with ATM was greater in extracts of cells overexpressing circLIFR, whereas the increased interaction was ablated when MSH2 was silenced (Fig. 5c and Fig. S4C). These results demonstrated that circLIFR augmented the binding of MutSα with ATM.

Previous studies have shown that MMR proteins contribute to the activation of apoptosis through p53-dependent and p53-independent mechanisms [13, 42, 43]. Thus, MMR-deficient cells exhibit variable defects in the induction of p53 and its two homologs p63 and p73, which are regulators of CDDP-induced apoptosis [44, 45]. Notably, p53 is the most frequently mutated gene in bladder cancer, while p63 and p73 are rarely mutated or deleted (Supplementary Table 4 and 5). Previous findings also confirmed that ATM played an important role in the regulation of p73-mediated apoptosis in response to CDDP [45]. Therefore, to further elucidate the pathway that mediated cell apoptosis, we examined the effect of circLIFR/MSH2 complex on ATM phosphorylation, as well as p63 and p73 expression. It showed that CDDP stimulated ATM phosphorylation and p73 expression in time-dependent manner, which were suppressed by MSH2 knockdown (Fig. 5d and Fig. S4D), and we observed that knockdown of MSH2 could impair both the extent and reaction rate of CDDP-induced ATM phosphorylation (Fig. 5d). Similarly, circLIFR silencing also inhibited the increase of ATM phosphorylation and p73 expression upon CDDP treatment (Fig. 5e and Fig. S4E). Moreover, enforced MSH2 expression, without exposure to CDDP, could significantly up-regulate phosphorylation of ATM and expression of p73, which were partially attenuated by circLIFR knockdown (Fig. 5f and Fig. S4F). In addition, we observed that circLIFR regulation of ATM phosphorylation and p73 expression were completely abrogated by MSH2 knockdown (Fig. 5g and Fig. S4G). Importantly, overexpression of circLIFR restored the ability of CDDP to induce ATM phosphorylation and p73 expression in CDDP resistant cells (Fig. 5h). These data supported the hypothesis that p73 could be positively regulated by circLIFR/MutSα complex through up-regulation of ATM phosphorylation.

To confirm whether the effects of MSH2 and circLIFR on cell apoptosis were mediated via p73, we conducted a series of rescue experiments. It showed that knockdown of P73 abolished MSH2-mediated increases of the basal level and CDDP-induced cell apoptosis (Fig. 5i and Fig. S4H). Similarly, circLIFR promotion of cell apoptosis was also completely reversed by p73 knockdown, both at the basal level and upon CDDP treatment (Fig. 5j and Fig. S4I). These results demonstrated that CircLIFR/MSH2 complex contributed to the CDDP sensitivity via MutSα/ATM-p73 axis in bladder cancer cells.

### CircLIFR is a potential therapeutic target to improve CDDP chemosensitivity in bladder cancer

**Fig. 6:**
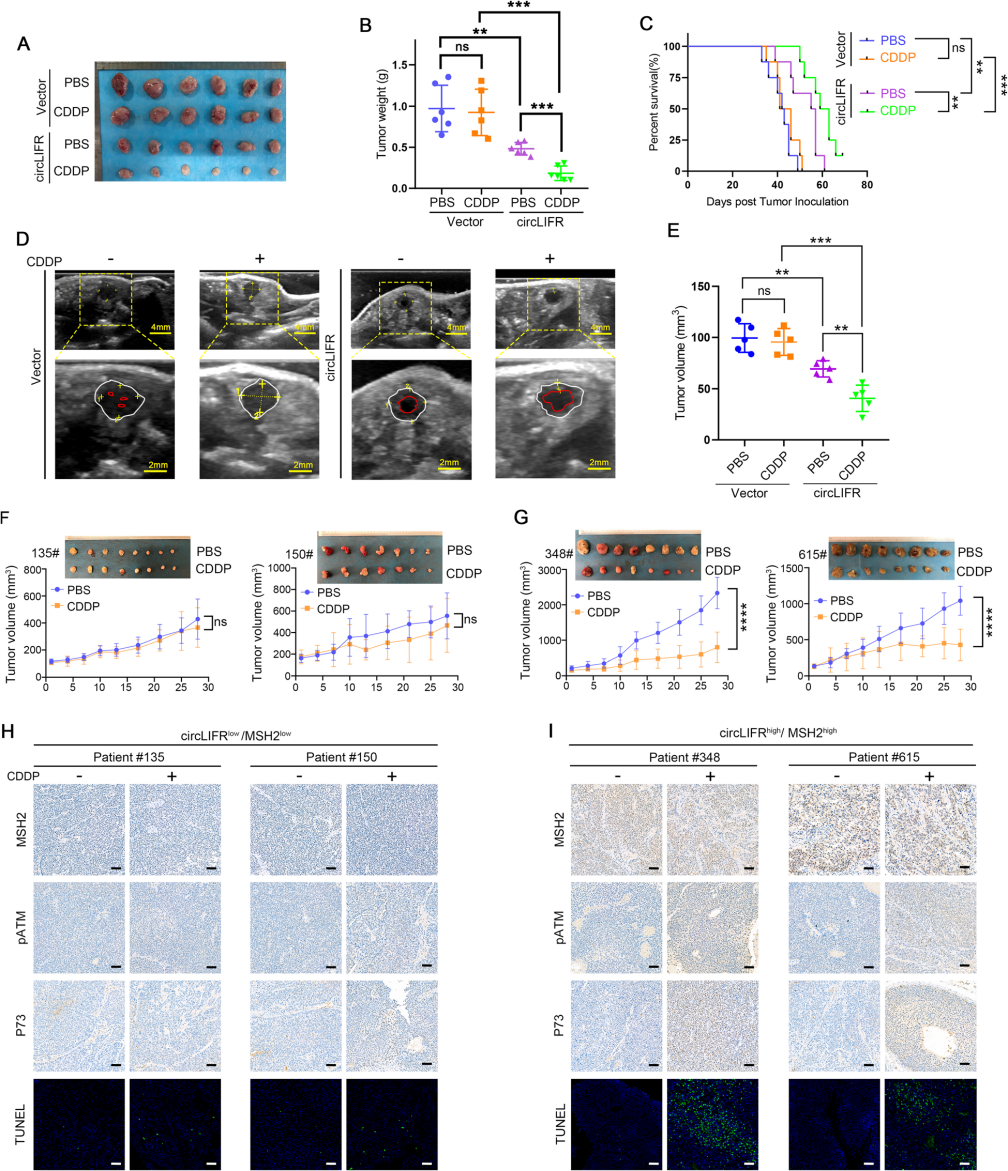
Biological implications of circLIFR in bladder cancer. **a**, **b** Response of T24-CDDP expressing vector or circLIFR xenografts to treatment with PBS or CDDP. The tumors on the 28th day of the treatments were shown (**a**); Graph showing the weight of tumors at the end of the treatment (**b**). Data were mean ± SD. ns, not significant, **P < 0.01, ***P < 0.001 (Student’s t-test). **c** Overall survival of T24-CDDP expressing vector or circLIFR xenografted mice treated with PBS or CDDP. *P*-value was calculated using a log-rank test. ns, not significant, **P < 0.01, ***P < 0.001. **d**, **e** The ultrasound images of orthotopic xenograft bladder tumor model established by T24-CDDP expressing vector or circLIFR, along with PBS or CDDP treatment. The low echo area with irregular surface between two lines represented the tumor and the echo free area inside the red line was the urine in urinary bladder. White line, the wall of urinary bladder; Red line, the convex surface of tumor toward the bladder lumen. Data were mean ± SD. ns, not significant, **P < 0.01, ***P < 0.001 (Student’s t-test). **f**, **g** Efficacy of CDDP therapy against the circLIFR^low^/MSH2^low^ (patient #135 and patient #150) and circLIFR^high^/MSH2^high^ (patient #348 and patient #615) PDX xenografts. Data were mean ± SD. ns, not significant, ****P < 0.0001 (Student’s t-test). **h**, **i**) Immunohistochemical images of MSH2, pATM, and p73 and TUNEL staining on circLIFR^low^/MSH2^low^ (patient #135 and patient #150) and circLIFR^high^/MSH2^high^ (patient #348 and patient #615) PDX xenografts. Scale bar: 50 μm

To determine whether circLIFR is an alternative therapeutic target which could improve CDDP-based therapy in CDDP-resistant tumors, T24-CDDP cells stably transfected with circLIFR or control vector were injected subcutaneously into BALB/c nude mice, followed by intrapleural PBS or CDDP treatment. Supporting the results obtained in vitro, as shown in Fig. 6 (A to C) and Fig. S5A, circLIFR strikingly decreased the tumor volumes and weights, prolonged survival, and weakened the CDDP resistance of T24-CDDP cells, whereas the administration of CDDP alone without the assistance of circLIFR overexpression could not retard tumor growth. Furthermore, in terms of that the subcutaneous model does not faithfully recapitulate the microenvironment of bladder cancer, we applied the orthotopic xenograft bladder tumor model along with PBS or CDDP treatment. Subsequent growth of bladder cancer was confirmed and monitored by urinary bladder ultrasound. Strikingly, we found that orthotopic transplantation of T24-CDDP cells with stable enforced expression of circLIFR displayed smaller tumor size and effectively resensitized CDDP-resistant cells to CDDP (Fig. 6d and e). These findings indicated that circLIFR could suppress tumor growth and be essential for governing CDDP chemotherapy efficacy even in CDDP-resistant bladder cancer cells in vivo.

To gain further insights into the potential therapeutic application of circLIFR and MSH2 on CDDP in patients, we used bladder cancer PDX model to explore the efficacy of CDDP. Based on the co-expression levels of circLIFR and MSH2 (Fig. S5B), we divided the clinical bladder cancer tissues into two groups, circLIFR^low^/MSH2^low^ group (patient #135 and patient #150) and circLIFR^high^/MSH2^high^ group (patient #348 and patient #615) (Fig. S5C). The PDX models of each patient were randomly separated and followed by intraperitoneal administration of PBS or CDDP, respectively (Fig. S5C). Of note, we found that the circLIFR^high^/MSH2^high^ group responded much better to CDDP than the circLIFR^low^/MSH2^low^ group (Fig. 6f and g). Consistent with these biological effects, a more intense TUNEL staining in circLIFR^high^/MSH2^high^ group compared with circLIFR^low^/MSH2^low^ group after administration of CDDP was appreciable (Fig. 6h and i). Importantly, IHC analysis revealed a more obvious improvement of ATM phosphorylation and p73 up-expression upon CDDP treatment in circLIFR^high^/MSH2^high^ group, compared with circLIFR^low^/MSH2^low^ group (Fig. 6h and i). Together, these data suggested that circLIFR and MSH2 status might be used as a stratification biomarker to select bladder cancer patients who may respond and benefit from CDDP treatment.

## Discussion

**Fig. 7:**
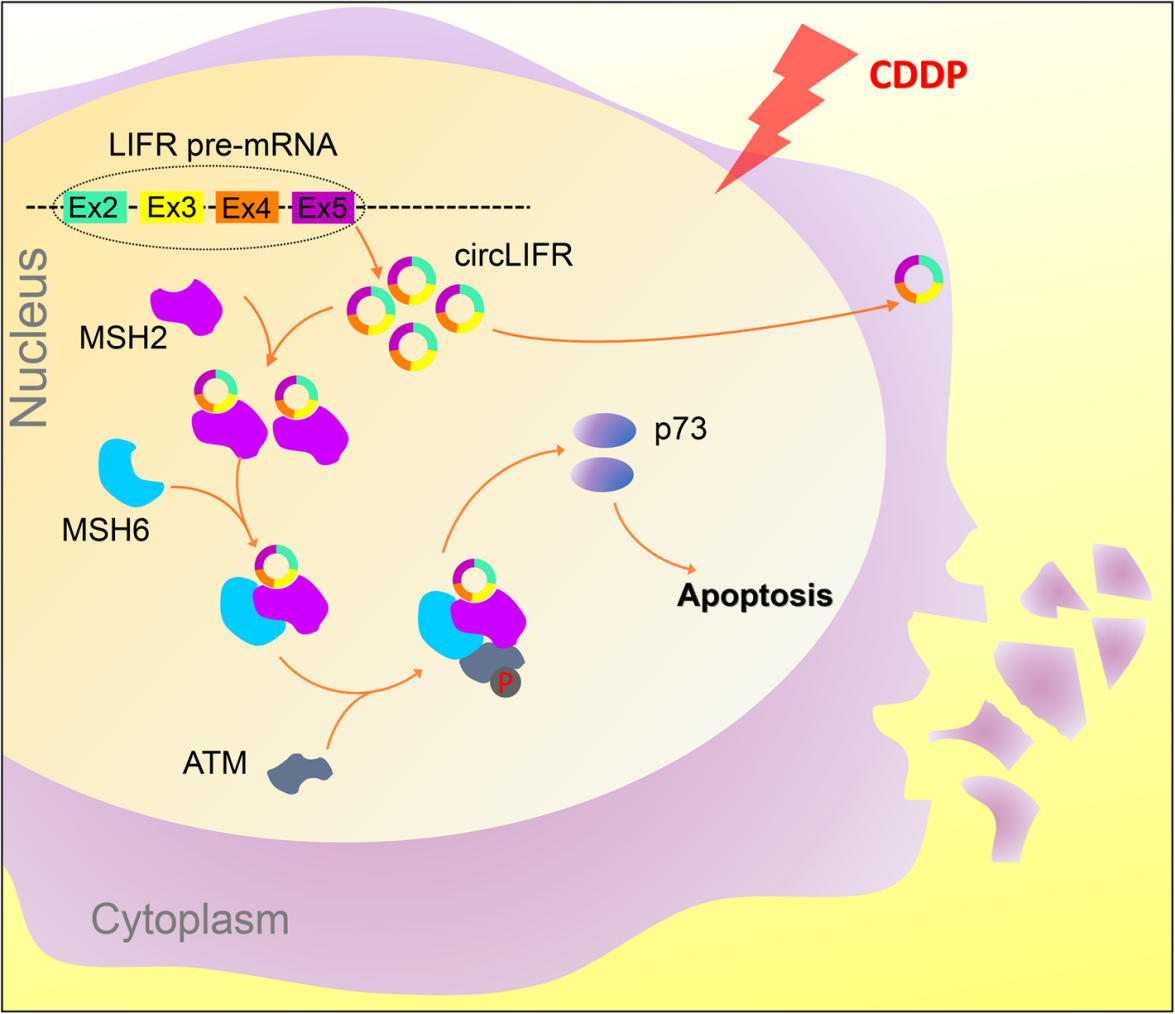
Schematic diagram of our hypothesis showing the effect of circLIFR/MutSα/ATM-p73 axis on CDDP chemosensitivity of bladder cancer. CircLIFR could bind to MSH2 protein in nucleus, where it augmented the binding of MutSα with ATM, and contributed to the CDDP sensitivity via pATM-p73 axis in bladder cancer

With a high rate of tumor heterogeneity, a large proportion of CDDP-treated bladder cancer patients experience therapeutic failure and tumor recurrence due to the acquisition of CDDP resistance, which is complex and poorly defined [1]. Understanding key pathway nodes that are crucial for driving resistance, especially genetic changes and/or epigenetic modifications, can provide a critical step toward circumventing cisplatin resistance in bladder cancer [46]. For instance, whole-exome sequencing and clonality analysis are performed to understand the relative contributions of different subclones and the effects of chemotherapy as a selective pressure in urothelial carcinoma [4]. Through an unbiased CRISPR screen in bladder cancer cells, MSH2 has 3 significantly CDDP resistant sgRNA constructs, and the importance of MSH2 is underscored by the fact that cancer cells lacking or expressing a low level of MSH2 lead to chemotherapy insensitivity and worse prognosis [13]. Herein, MSH2 was identified to interact with circLIFR by mass spectrometry analysis. Mechanistically, circLIFR bound and synergized with MSH2 protein, which augmented the interaction between MutSα and ATM, to up-regulate p73 expression, ultimately contributing to attenuate bladder cancer growth and cellular tolerance to CDDP (Fig. 7). Moreover, bladder cancer cell lines xenograft models and PDX models provided preliminary assessment of the response to CDDP therapy with different levels of circLIFR and MSH2. These findings uncovered circLIFR and MSH2 as tumor suppressors involving novel layers of CDDP chemotherapy regulation and provided further evidence that circRNAs are fundamental players in bladder cancer progression.

It is evident that circRNAs are prevalent genes with frequently exquisite regulation and recognized as promising candidates for the identification of additional layers of gene expression control in human tissues [16]. CircRNAs have been well characterized in a variety of human diseases, including cancer, neurological disorders, cardiovascular diseases and metabolic disorders [16, 18, 20]. Recently, it has also been reported that circRNAs regulate CDDP chemotherapy by sponging miRNAs. Specifically, circAKT3, which localizes to and functions in the cytoplasm, modulates CDDP sensitivity by sponging miR-198 that suppresses PIK3R1 expression in gastric cancer [47]. Circular RNA Cdr1as sensitizes bladder cancer to CDDP by upregulating APAF1 expression through miR-1270 inhibition [48]. In the present study, we identified that circLIFR was a bona fide circRNA and was mainly localized in the nucleus. Gain- and loss-of-function studies demonstrated that circLIFR could increase cell apoptosis and sensitize cells to CDDP treatment. Although related circRNAs of chemotherapy regulation are now documented, circLIFR was distinguished by its role in influencing chemosensitivity by combining and synergizing with MSH2, a well-established key protein regulating CDDP chemotherapy. More importantly, our results suggested that circRNAs, as regulatory factors, could bind to key effector proteins that influenced chemotherapy, providing a novel model of chemotherapy regulation mechanism. Furthermore, circLIFR could act as potent chemosensitizer in the nucleus, suggesting new ideas for clinical transformation. In our experiments, we ruled out the function of circLIFR that acted as miRNA sponge, and we found that circLIFR performed its protein binding role in the nucleus. However, the potential abilities to translate peptides of circLIFR in cytoplasm still need to be further clarified.

MSH2 and MSH6 proteins are divided into five conserved domains, among which the C-terminal is ATPase domain [10]. Moreover, ATPase domain of MSH2 exhibits multiple interaction sites with MSH6 in MutSα [10]. In this paper, RNA pulldown and RIP analysis demonstrated that circLIFR interacted with the ATPase domain of MSH2 and resulted in promoting the assembly of MutSα, which indicated that circLIFR might favour protein folding and act as a molecular chaperone. Our results also showed that circLIFR mediated MSH2-dependent apoptosis through MutSα. How the MutSα complex participates in the apoptotic signaling cascade remains subject to debate, with two competing hypothesis dominating academic contention [49, 50]. The “futile repair cycle” hypothesis entails repetitive repair attempts of a DNA strand containing lesions. DNA damage signaling is triggered by abortive repair attempts and persistent DNA damage. Because of this, functional repair activity of the MMR proteins is a prerequisite for this proposed mechanism [49]. Conversely, the “direct signaling” hypothesis propounds a dual functionality for MutSα complex: a “pro-repair” conformation in which DNA repair is promoted, and an alternative “pro-apoptotic” conformation in which the protein abandons its repair function and instead activates apoptosis response [51]. Herein, based on the findings that circLIFR and MSH2 were sufficient to mediate apoptosis in the absence of DNA damage, we speculated that circLIFR induced a MutSα “pro-apoptotic” conformation to initiate MSH2-dependent cell death. Combined with prior published studies where a small molecule, reserpine, capable of binding MSH2 can stimulate the conformational change and initiate the same cellular response as DNA damage [51], our results supported the “direct signaling” hypothesis. Nevertheless, we cannot rule out the possibility that the “futile repair cycle” hypothesis is participated in the apoptosis regulated by circLIFR/MSH2 and CDDP. Furthermore, given MSH2 can ensure genetic stability by correcting DNA biosynthetic errors [7], it is yet undetermined whether circLIFR plays a role in DNA mismatch repair. The topological structure of circLIFR/MutSα complex is still need to be further characterized, which may reveal detailed features of this interaction and find out whether circLIFR plays an important role in the conformational change of MutSα.

MutSα forms a complex with ATM, which is the central checkpoint kinases in signaling DNA damage [41]. As predicted, CDDP and circLIFR augmented the formation of MutSα/ATM complex, which in turn, phosphorylated ATM. Although ATM signaling in breast cancer or cervical cancer leads to doxorubicin or MNNG resistance [52, 53], cell-death to etoposide or curcumin chemotherapy may arise from ATM signaling in osteosarcoma or pancreatic cancer [54, 55], highlighting the contextual importance of individual studies where activation of ATM may have divergent roles. More importantly, previous results suggest that ATM signaling, which stabilizes p73, is one of the main apoptotic pathways in response to CDDP [45]. Likewise, ATM-p73 axis regulated by circLIFR/MutSα was an important determinant of chemotherapy susceptibility in bladder cancer. Moreover, p73 does not appear to be inactivated during malignant transformation, whereas p53 is frequently mutated [14, 42, 56]. Hence, a therapeutic that activates the circLIFR /MutSα/ATM-p73 axis-dependent cell-death pathway might be advantageous as it would eliminate the requirement for functional p53. However, whether the mechanism might exist in other cell types other than bladder cancer cells needs to be further investigated.

## Conclusions

In summary, our work provides a proof of concept for circRNAs as molecular regulators of MMR proteins and of key cellular functions relevant to chemotherapy for bladder cancer. These findings implicate a circLIFR/MutSα/ATM-p73 axis in the progression of bladder cancer and the role of chemotherapy resistance. Therefore, the mechanistic characterization of circLIFR and its functional crosstalk with MSH2 may help to pave the way to develop bladder cancer chemotherapies that target MSH2 and its interaction with circLIFR.

## Supplementary Information

**Additional file 1: Fig. S1.** Identification and distribution of circLIFR. (A, B) The expression of pLIFR and mLIFR was detected by qRT-PCR in 79 pairs of bladder cancer and paired adjacent normal bladder tissues. Data were mean ± SD. ns, not significant (Student’s t-test). (C) Kaplan-Meier curves of OS in bladder cancer patients. Patients were grouped by the median mLIFR expression. *P*-value was calculated using a log-rank test. (D) Reverse transcription was performed by random 6 mers and oligo dT primer, respectively. Then, the relative RNA levels of circLIFR and mLIFR were analyzed by qRT-PCR. Data were mean ± SD. ns, not significant, ***P < 0.001 (Student’s t-test). (E) The relative RNA levels of circLIFR and mLIFR were analyzed by qRT-PCR after treatment with Actinomycin D at the indicated time points in UMUC3 cells. (F) Identification of circLIFR cytoplasmic and nuclear distribution by qRT-PCR analysis in UMUC3 cells. GAPDH and U1 were applied as positive controls in the cytoplasm and nucleus, respectively (*n* = 3). Western blots of total cell lysates (T), cytosolic extracts (C) and nuclear extracts (N) with α-tubulin as a cytosolic marker, histone H3 as a nuclear marker. (G) Identification of circLIFR cytoplasmic and nuclear distribution by FISH in UMUC3 cells. 18S and U6 were applied as positive controls in the cytoplasm and nucleus, respectively; circLIFR, 18S, and U6 probes were labeled with Cy3; nuclei were stained with DAPI

**Additional file 2: Fig. S2.** CircLIFR binds to MSH2 protein. (A) RIP analysis was carried out using anti-AGO2 or IgG antibodies. circLIFR, CDR1as, and U6 levels in the samples were quantified using qRT-PCR. CDR1as and U6 were applied as positive and negative controls that interacting with AGO2, respectively. Data were mean ± SD. ns, not significant, **P < 0.01 (Student’s t-test). (B) Schematic of biotin-labeled sense or antisense circLIFR probes and efficient pull-down of circLIFR in T24 and UMUC3 cells. Data were mean ± SD. ****P < 0.0001 (Student’s t-test)

**Additional file 3: Fig. S3.** MSH2 and circLIFR can improve CDDP chemosensitivity. (A) Determination of MSH2 protein levels in T24 and UMUC3 cells transfected with scramble, shMSH2#1, or shMSH2#2. (B, C) UMUC3 cells were stably transfected with scramble, shMSH2#1, or shMSH2#2 vector. After UMUC3 cells were treated for 36 h in the absence or presence of 3 μM CDDP, apoptosis was measured by Annexin-V plus PI staining and FACS analysis. Data were mean ± SD. ***P < 0.001, ****P < 0.0001 (Student’s t-test). (D) Determination of IC50 values for CDDP treatment 24 h in UMUC3 cells which were stably transfected with scramble, shMSH2#1, shMSH2#2, mock, or MSH2 vector. (E) Efficient knockdown of circLIFR in T24 cells. Data were mean ± SD. ns, not significant, ***P < 0.001, ****P < 0.0001 (Student’s t-test). (F) Effect of overexpression of circLIFR on mLIFR expression. Data were mean ± SD. ns, not significant (Student’s t-test). (G, H) UMUC3 cells were stably transfected with scramble, sh-circLIFR#2 vector. After T24 cells were treated for 36 h in the absence or presence of 3 μM CDDP, apoptosis was measured by Annexin-V plus PI staining and FACS analysis. Data were mean ± SD. ***P < 0.001, ****P < 0.0001 (Student’s t-test). (I) Determination of IC50 values for CDDP treatment 24 h in UMUC3 cells which were stably transfected with scramble, sh-circLIFR#2, vector, or circLIFR. (J) circLIFR levels and MSH2 mRNA/protein levels between T24-CDDP and parental T24 cells. Data were mean ± SD. ns, not significant (Student’s t-test)

**Additional file 4: Fig. S4.** CircLIFR/MSH2 complex contributes to the CDDP sensitivity via MutSα/ATM-p73 axis in bladder cancer cells. (A) The relative RNA levels were analyzed by qRT-PCR in T24 and UMUC3 cells stably transfected with vector or circLIFR. Western blot analysis with the indicated antibodies in T24 cells and UMUC3 stably transfected with vector or circLIFR. Data were mean ± SD. ns, not significant, ***P < 0.001 (Student’s t-test). (B) The relative RNA levels were analyzed by qRT-PCR in T24 and UMUC3 cells stably transfected with mock or MSH2. Western blot analysis with the indicated antibodies in T24 cells and UMUC3 stably transfected with mock or MSH2. Data were mean ± SD. ns, not significant, ***P < 0.001 (Student’s t-test). (C) Interaction between MSH2, MSH6, MSH3, and ATM in UMUC3 cells stably transfected with vector or circLIFR, and those cotransfected with scramble, or shMSH2#1. Co-IP experiments with anti-MSH2 antibody were performed, and the precipitate was detected by western blot with the antibodies against MSH2, MSH6, MSH3, ATM, and GAPDH. (D) UMUC3 cells, which were stably transfected with scramble, shMSH2#1, or shMSH2#2, were treated with 3 μM CDDP for the indicated time. Whole cell lysates were collected for western blot analysis of MSH2, MSH6, ATM, pATM, p63, p73, and GAPDH. (E) UMUC3 cells, which were stably transfected with scramble, or sh-circLIFR#2, were treated with 3 μM CDDP for the indicated time. Whole cell lysates were collected for western blot analysis with the indicated antibodies. (F) Western blot analysis with the indicated antibodies in UMUC3 cells stably transfected with vector or MSH2, and those cotransfected with scramble, or sh-circLIFR#2. (G) Western blot analysis with the indicated antibodies in UMUC3 cells stably transfected with vector or circLIFR, and those cotransfected with scramble, shMSH1#1, or shMSH1#2. (H) Western blot analysis with the indicated antibodies in T24 cells stably transfected with vector or MSH2, and those cotransfected with scramble, or sh-p73. (I) Western blot analysis with the indicated antibodies in T24 cells stably transfected with vector or circLIFR, and those cotransfected with scramble, or sh-p73.

**Additional file 5: Fig. S5.** Biological implications of circLIFR in bladder cancer. (A) Tumor growth curve showing the response of T24-CDDP expressing vector or circLIFR xenografts to treatment with PBS or CDDP. Data were mean ± SD. ns, not significant, ***P < 0.001, ****P < 0.0001 (Student’s t-test). (B) The relative RNA levels were analyzed by qRT-PCR in patient #135, patient #150, patient #348, and patient #615. GAPDH was used as internal control. Data were mean ± SD. ns, not significant, ****P < 0.0001 (Student’s t-test). (C) Schematic diagram of the treatment regimen with PBS, or CDDP

**Additional file 6: Supplementary Table S1.**

**Additional file 7: Supplementary Table S2.**

**Additional file 8: Supplementary Table S3.**

**Additional file 9: Supplementary Table S4.**

**Additional file 10: Supplementary Table S5.**

## Data Availability

The datasets supporting the conclusions of this article are included within the article and its supplementary files.

## Abbreviations

CDDP: Cisplatin
circRNAs: circular RNAs
MIBC: Muscle-invasive bladder cancer
MMR: DNA mismatch repair
MSH2: MutS homolog 2
MLH1: MutL homolog 1
HNPCC: Hereditary nonpolyposis colorectal cancer
OS: Overall survival
T24-CDDP: Cisplatin-resistant variants of T24
RIP: RNA Immunoprecipitation
FISH: Fluorescent in situ hybridization
GSEA: Gene set enrichment analysis
CCK-8: Cell Counting Kit-8
PDX: Patient-derived xenograft
IHC: Immunohistochemistry
TUNEL: Terminal deoxyribonucleotide transferase-mediated nick-end labeling
pLIFR: pre-mRNA of LIFR
mLIFR: mRNA of LIFR
shRNAs: small hairpin RNAs
