# Supplementary figures and images for: CircLIFR synergizes with MSH2 to attenuate chemoresistance via MutSα/ATM-p73 axis in bladder cancer
Source: Mol Cancer. 2021 Apr 19;20:70. doi: 10.1186/s12943-021-01360-4 (PMC8054397; doi:10.1186/s12943-021-01360-4)

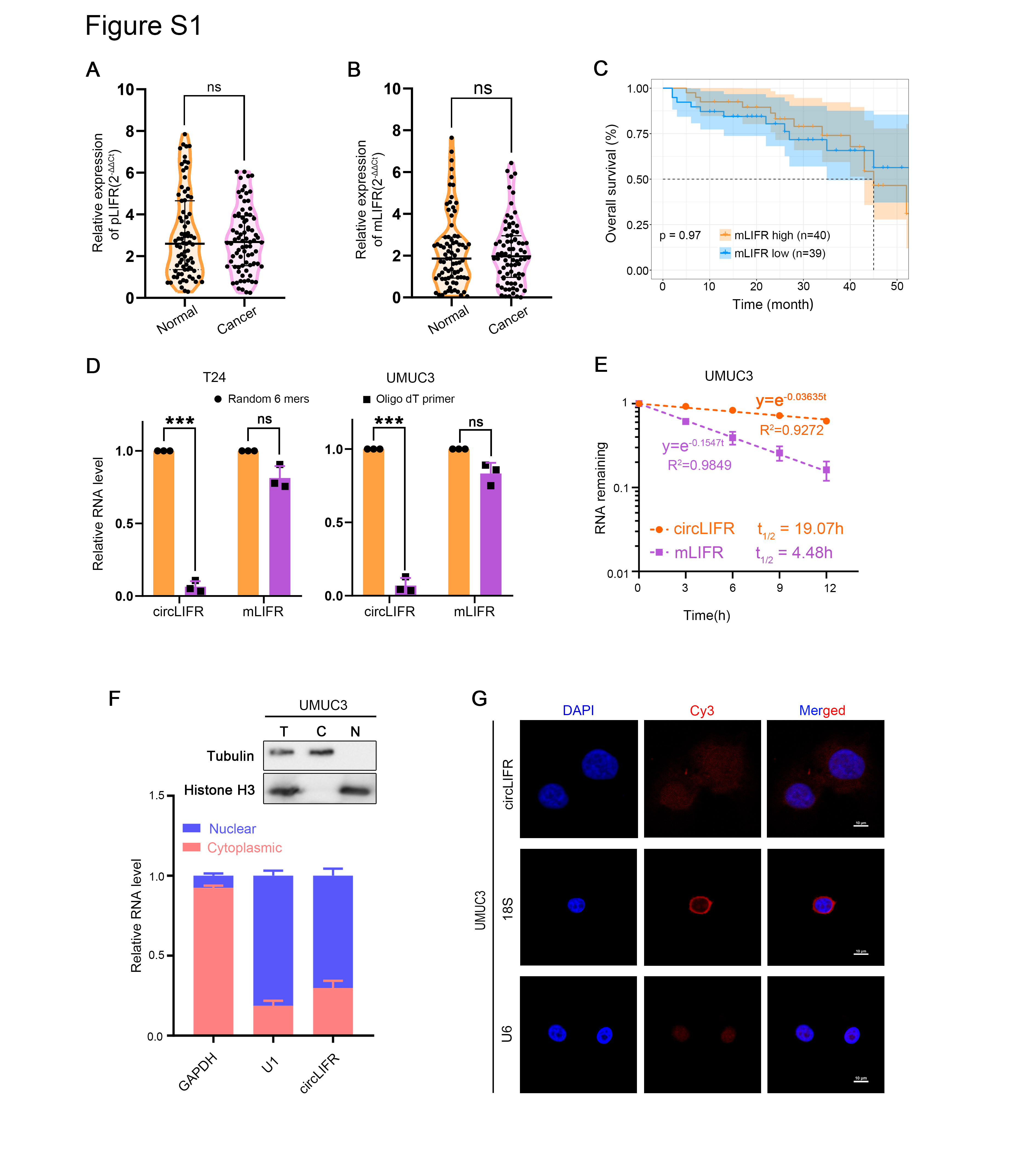

Supplement: Supplementary file 1 — Additional file 1: Fig. S1. Identification and distribution of circLIFR. (A, B) The expression of pLIFR and mLIFR was detected by qRT-PCR in 79 pairs of bladder cancer and paired adjacent normal bladder tissues. Data were mean ± SD. ns, not significant (Student’s t-test). (C) Kaplan-Meier curves of OS in bladder cancer patients. Patients were grouped by the median mLIFR expression. P-value was calculated using a log-rank test. (D) Reverse transcription was performed by random 6 mers and oligo dT primer, respectively. Then, the relative RNA levels of circLIFR and mLIFR were analyzed by qRT-PCR. Data were mean ± SD. ns, not significant, ***P < 0.001 (Student’s t-test). (E) The relative RNA levels of circLIFR and mLIFR were analyzed by qRT-PCR after treatment with Actinomycin D at the indicated time points in UMUC3 cells. (F) Identification of circLIFR cytoplasmic and nuclear distribution by qRT-PCR analysis in UMUC3 cells. GAPDH and U1 were applied as positive controls in the cytoplasm and nucleus, respectively (n = 3). Western blots of total cell lysates (T), cytosolic extracts (C) and nuclear extracts (N) with α-tubulin as a cytosolic marker, histone H3 as a nuclear marker. (G) Identification of circLIFR cytoplasmic and nuclear distribution by FISH in UMUC3 cells. 18S and U6 were applied as positive controls in the cytoplasm and nucleus, respectively; circLIFR, 18S, and U6 probes were labeled with Cy3; nuclei were stained with DAPI [file 12943_2021_1360_MOESM1_ESM.jpg]

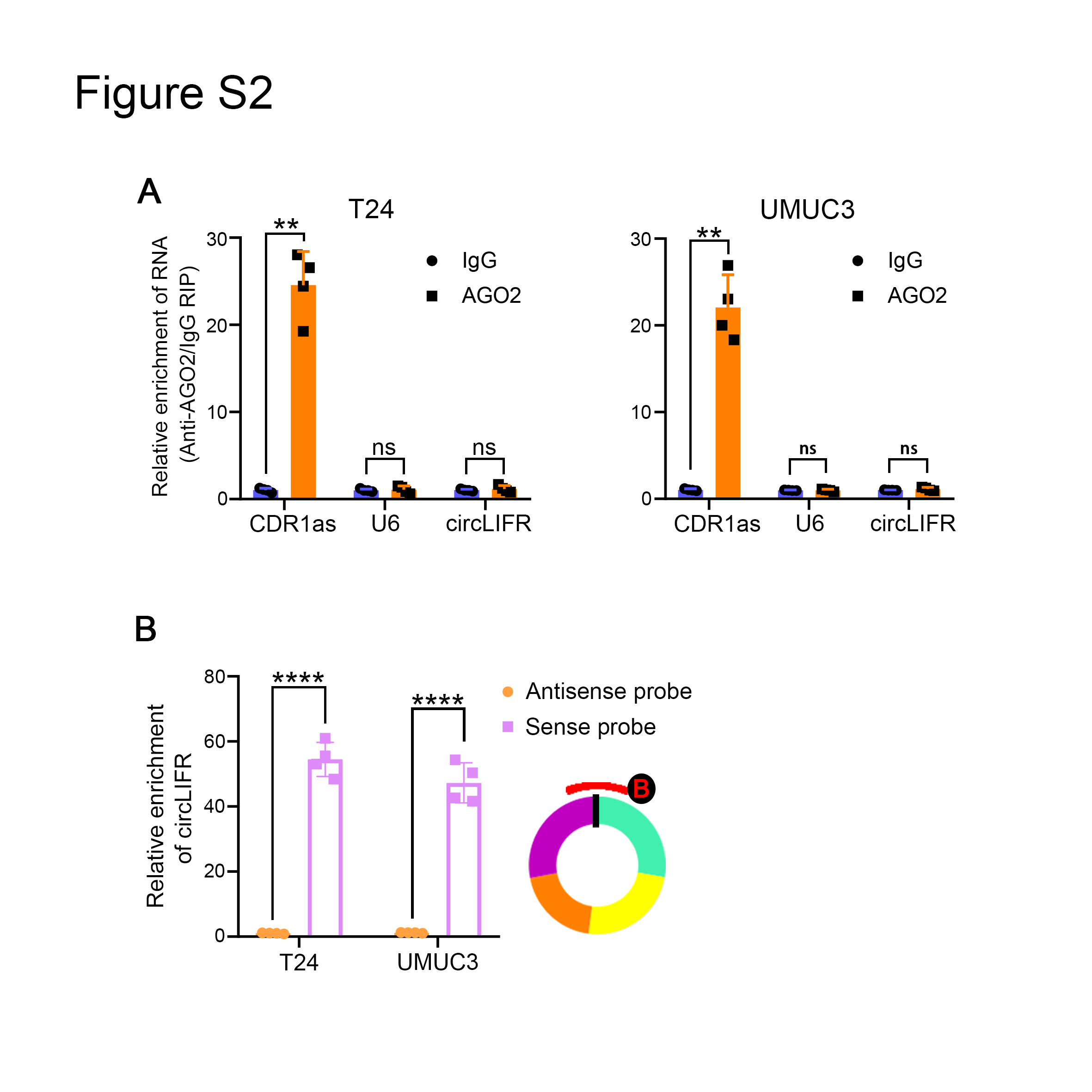

Supplement: Supplementary file 2 — Additional file 2: Fig. S2. CircLIFR binds to MSH2 protein. (A) RIP analysis was carried out using anti-AGO2 or IgG antibodies. circLIFR, CDR1as, and U6 levels in the samples were quantified using qRT-PCR. CDR1as and U6 were applied as positive and negative controls that interacting with AGO2, respectively. Data were mean ± SD. ns, not significant, **P < 0.01 (Student’s t-test). (B) Schematic of biotin-labeled sense or antisense circLIFR probes and efficient pull-down of circLIFR in T24 and UMUC3 cells. Data were mean ± SD. ****P < 0.0001 (Student’s t-test) [file 12943_2021_1360_MOESM2_ESM.jpg]

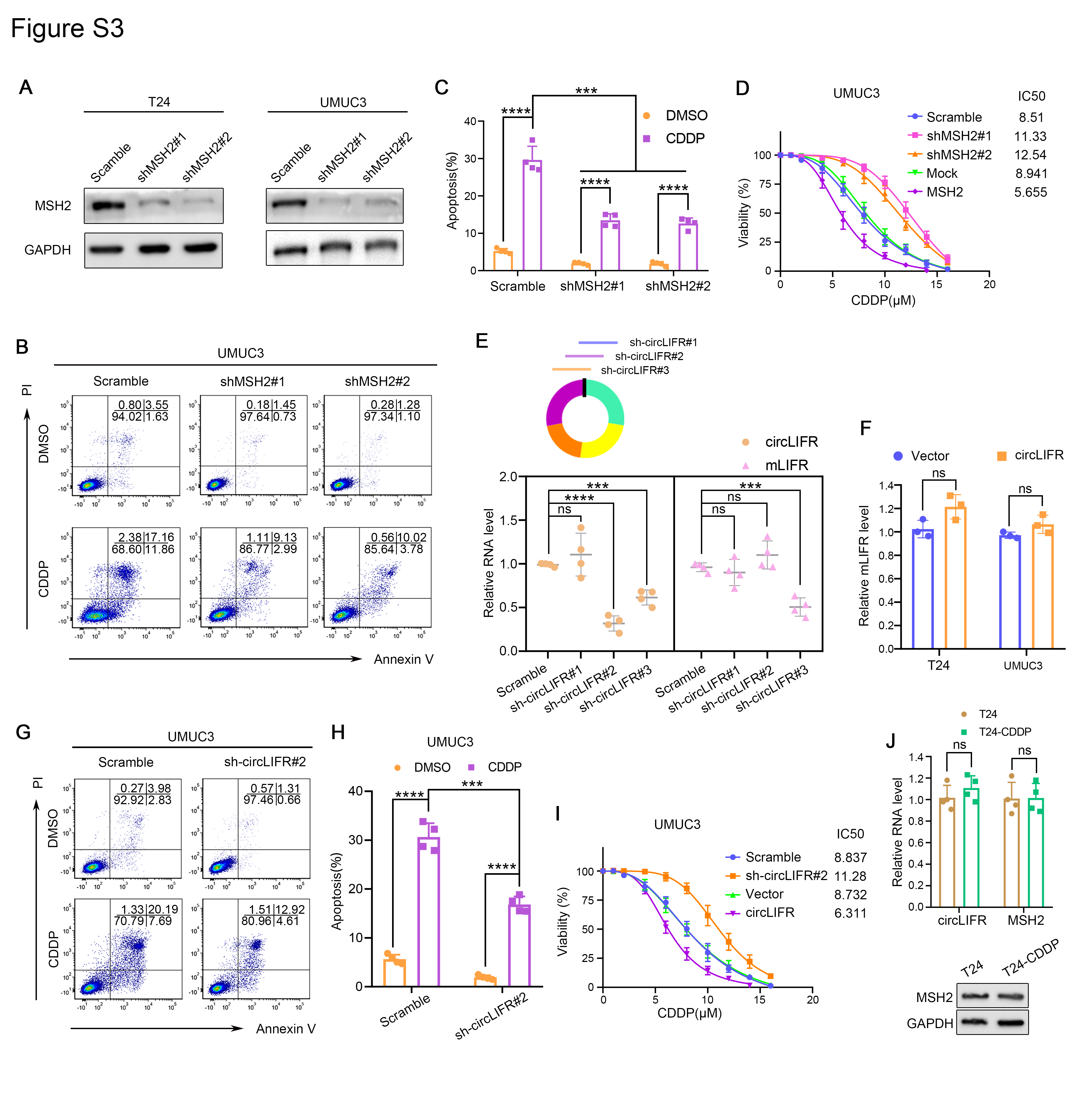

Supplement: Supplementary file 3 — Additional file 3: Fig. S3. MSH2 and circLIFR can improve CDDP chemosensitivity. (A) Determination of MSH2 protein levels in T24 and UMUC3 cells transfected with scramble, shMSH2#1, or shMSH2#2. (B, C) UMUC3 cells were stably transfected with scramble, shMSH2#1, or shMSH2#2 vector. After UMUC3 cells were treated for 36 h in the absence or presence of 3 μM CDDP, apoptosis was measured by Annexin-V plus PI staining and FACS analysis. Data were mean ± SD. ***P < 0.001, ****P < 0.0001 (Student’s t-test). (D) Determination of IC50 values for CDDP treatment 24 h in UMUC3 cells which were stably transfected with scramble, shMSH2#1, shMSH2#2, mock, or MSH2 vector. (E) Efficient knockdown of circLIFR in T24 cells. Data were mean ± SD. ns, not significant, ***P < 0.001, ****P < 0.0001 (Student’s t-test). (F) Effect of overexpression of circLIFR on mLIFR expression. Data were mean ± SD. ns, not significant (Student’s t-test). (G, H) UMUC3 cells were stably transfected with scramble, sh-circLIFR#2 vector. After T24 cells were treated for 36 h in the absence or presence of 3 μM CDDP, apoptosis was measured by Annexin-V plus PI staining and FACS analysis. Data were mean ± SD. ***P < 0.001, ****P < 0.0001 (Student’s t-test). (I) Determination of IC50 values for CDDP treatment 24 h in UMUC3 cells which were stably transfected with scramble, sh-circLIFR#2, vector, or circLIFR. (J) circLIFR levels and MSH2 mRNA/protein levels between T24-CDDP and parental T24 cells. Data were mean ± SD. ns, not significant (Student’s t-test) [file 12943_2021_1360_MOESM3_ESM.jpg]

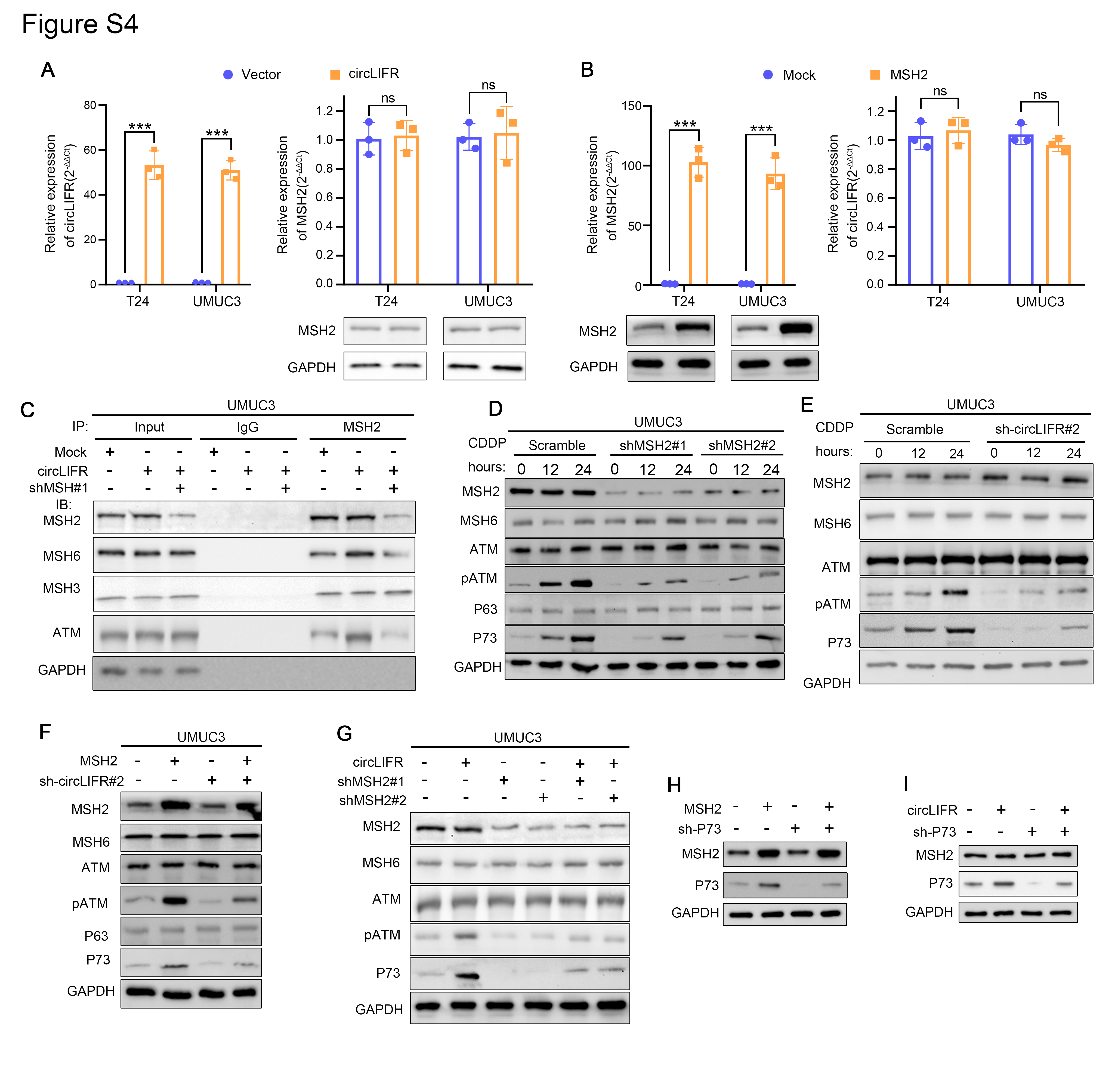

Supplement: Supplementary file 4 — Additional file 4: Fig. S4. CircLIFR/MSH2 complex contributes to the CDDP sensitivity via MutSα/ATM-p73 axis in bladder cancer cells. (A) The relative RNA levels were analyzed by qRT-PCR in T24 and UMUC3 cells stably transfected with vector or circLIFR. Western blot analysis with the indicated antibodies in T24 cells and UMUC3 stably transfected with vector or circLIFR. Data were mean ± SD. ns, not significant, ***P < 0.001 (Student’s t-test). (B) The relative RNA levels were analyzed by qRT-PCR in T24 and UMUC3 cells stably transfected with mock or MSH2. Western blot analysis with the indicated antibodies in T24 cells and UMUC3 stably transfected with mock or MSH2. Data were mean ± SD. ns, not significant, ***P < 0.001 (Student’s t-test). (C) Interaction between MSH2, MSH6, MSH3, and ATM in UMUC3 cells stably transfected with vector or circLIFR, and those cotransfected with scramble, or shMSH2#1. Co-IP experiments with anti-MSH2 antibody were performed, and the precipitate was detected by western blot with the antibodies against MSH2, MSH6, MSH3, ATM, and GAPDH. (D) UMUC3 cells, which were stably transfected with scramble, shMSH2#1, or shMSH2#2, were treated with 3 μM CDDP for the indicated time. Whole cell lysates were collected for western blot analysis of MSH2, MSH6, ATM, pATM, p63, p73, and GAPDH. (E) UMUC3 cells, which were stably transfected with scramble, or sh-circLIFR#2, were treated with 3 μM CDDP for the indicated time. Whole cell lysates were collected for western blot analysis with the indicated antibodies. (F) Western blot analysis with the indicated antibodies in UMUC3 cells stably transfected with vector or MSH2, and those cotransfected with scramble, or sh-circLIFR#2. (G) Western blot analysis with the indicated antibodies in UMUC3 cells stably transfected with vector or circLIFR, and those cotransfected with scramble, shMSH1#1, or shMSH1#2. (H) Western blot analysis with the indicated antibodies in T24 cells stably transfected with vector or MSH2, [file 12943_2021_1360_MOESM4_ESM.jpg]

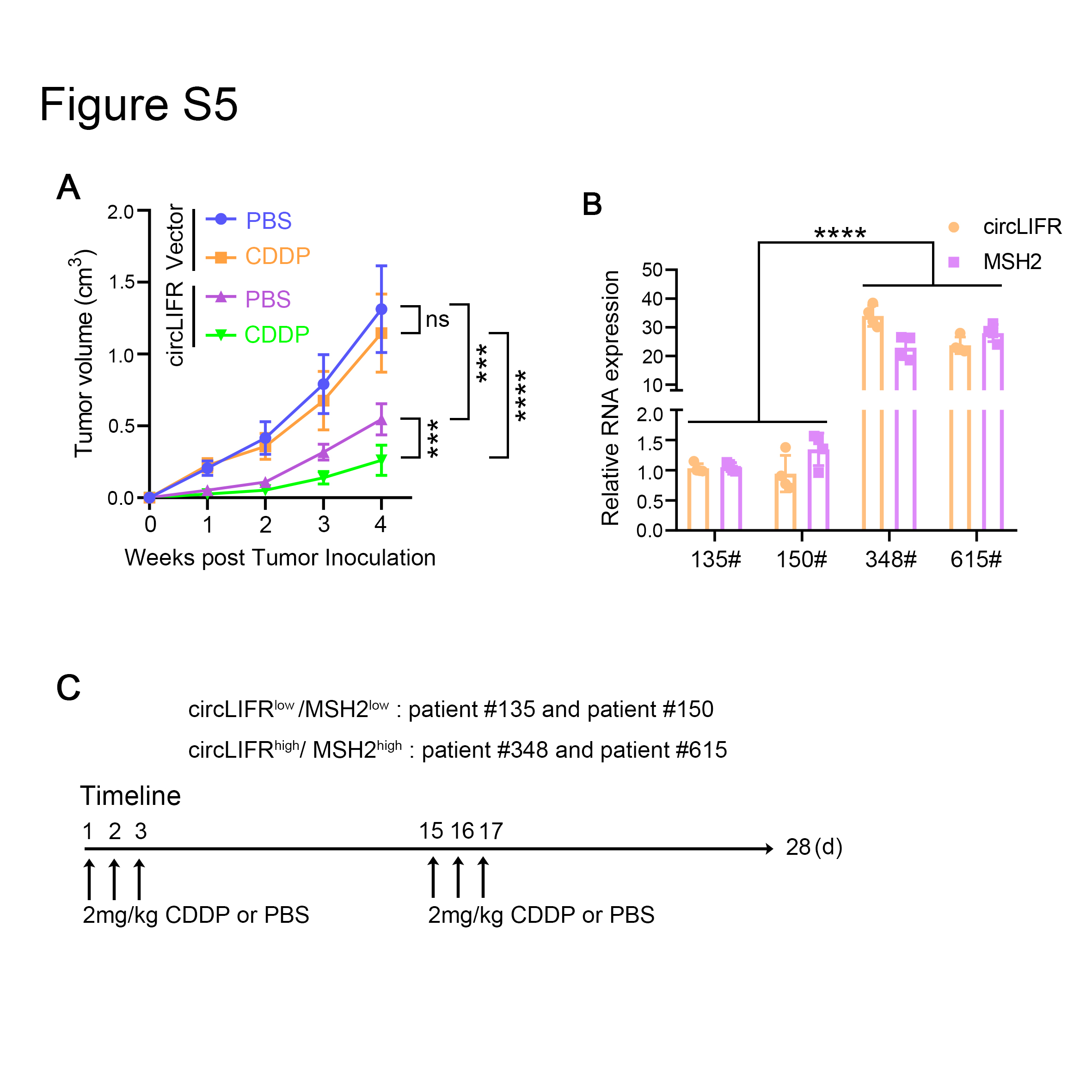

Supplement: Supplementary file 5 — Additional file 5: Fig. S5. Biological implications of circLIFR in bladder cancer. (A) Tumor growth curve showing the response of T24-CDDP expressing vector or circLIFR xenografts to treatment with PBS or CDDP. Data were mean ± SD. ns, not significant, ***P < 0.001, ****P < 0.0001 (Student’s t-test). (B) The relative RNA levels were analyzed by qRT-PCR in patient #135, patient #150, patient #348, and patient #615. GAPDH was used as internal control. Data were mean ± SD. ns, not significant, ****P < 0.0001 (Student’s t-test). (C) Schematic diagram of the treatment regimen with PBS, or CDDP [file 12943_2021_1360_MOESM5_ESM.jpg]
