# Supplementary material for: CircLIFR synergizes with MSH2 to attenuate chemoresistance via MutSα/ATM-p73 axis in bladder cancer
Source: Mol Cancer. 2021 Apr 19;20:70. doi: 10.1186/s12943-021-01360-4 (PMC8054397; doi:10.1186/s12943-021-01360-4)
Supplement: Supplementary file 9 — Additional file 9: Supplementary Table S4. [file 12943_2021_1360_MOESM9_ESM.docx]

Supplementary Table 4. TP53, TP63, and TP73 mutation in T24 and UMUC3 cells (data from CCLE)

| Hugo Symbol | Tumor Sample Barcode | Variant Type | Variant Classification | Protein Change |
| --- | --- | --- | --- | --- |
| TP53 | T24_URINARY_TRACT | SNP | Nonsense_Mutation | p.Y126* |
| TP53 | UMUC3_URINARY_TRACT | SNP | Missense_Mutation | p.F113C |
| TP63 | T24_URINARY_TRACT | NONE | NONE | NONE |
| TP63 | UMUC3_URINARY_TRACT | NONE | NONE | NONE |
| TP73 | T24_URINARY_TRACT | NONE | NONE | NONE |
| TP73 | UMUC3_URINARY_TRACT | NONE | NONE | NONE |
