# Supplementary material for: CircLIFR synergizes with MSH2 to attenuate chemoresistance via MutSα/ATM-p73 axis in bladder cancer
Source: Mol Cancer. 2021 Apr 19;20:70. doi: 10.1186/s12943-021-01360-4 (PMC8054397; doi:10.1186/s12943-021-01360-4)
Supplement: Supplementary file 10 — Additional file 10: Supplementary Table S5. [file 12943_2021_1360_MOESM10_ESM.docx]

| Gene Symbol | Project | Tumor Subtype | Number of SSM-tested donors in the current project | Mutations | Percentage of donors with the current gene |
| --- | --- | --- | --- | --- | --- |
| TP53 | Bladder Urothelial Cancer - TCGA, US | Invasive Urothelial Bladder cancer | 411 | 139 | 0.47 |
| TP53 | Bladder Cancer - CN | Urothelial carcinoma | 103 | 26 | 0.27 |
| TP63 | Bladder Urothelial Cancer - TCGA, US | Invasive Urothelial Bladder cancer | 411 | 24 | 0.06 |
| TP63 | Bladder Cancer - CN | Urothelial carcinoma | 103 | 3 | 0.03 |
| TP73 | Bladder Urothelial Cancer - TCGA, US | Invasive Urothelial Bladder cancer | 411 | 8 | 0.02 |
| TP73 | Bladder Cancer - CN | Urothelial carcinoma | 103 | 1 | 0.01 |

Supplementary Table 5. TP53, TP63, and TP73 mutation in bladder cancer tissue (data from ICGC)
